# Induced collagen type‐I secretion by hepatocytes of the melanoma liver metastasis is associated with a reduction in tumour‐infiltrating lymphocytes

**DOI:** 10.1002/ctm2.70067

**Published:** 2024-11-04

**Authors:** Shodai Mizuno, Matias A. Bustos, Yoshinori Hayashi, Kodai Abe, Satoru Furuhashi, Yalda Naeini, Xiaowei Xu, Anton J Bilchik, Dave S. B. Hoon

**Affiliations:** ^1^ Department of Translational Molecular Medicine Saint John's Cancer Institute (SJCI) at Providence Saint John's Health Center (SJHC) Santa Monica California USA; ^2^ Department of Surgical Pathology at Providence SJHC Santa Monica California USA; ^3^ Abramson Cancer Center, Perelman School of Medicine University of Pennsylvania Philadelphia Pennsylvania USA; ^4^ Department of Gastrointestinal and Hepatobiliary Surgery, Providence SJHC Santa Monica California USA; ^5^ Department of Genome Sequencing Center SJCI, Providence SJHC Santa Monica California USA

**Keywords:** collagen, melanoma liver metastasis, normal hepatocytes, spatial analysis, tumour‐microenvironment

## Abstract

**Background:**

Overall patients with melanoma liver metastasis (MLiM) have a dismal prognosis and poor responses to the standard of care treatment. Understanding the role of the tumour microenvironment (TME) is critical for discovering better strategies to overcome intrinsic therapy resistance in MLiM. The aim was to understand the crosstalk signalling pathways between hepatocytes and metastatic melanoma cells in the TME of MLiM.

**Methods:**

Hepatocytes and melanoma tumour cells of MLiM were assessed using transcriptomic NanoString GeoMx digital spatial profiling (NGDSP) assay. Functional assays were performed using normal hepatocytes and MLiM‐derived cell lines. Validation was performed using multiplex immunofluorescence.

**Results:**

In NGDSP analysis adjacent normal hepatocytes (ANH) had higher CXCR4 and COL1A1/2 levels than distant normal hepatocytes (DNH), while melanoma cells had higher TNF‐α levels. In vitro, MLiM cell lines released TNF‐α which upregulated CXCR4 and CXCL12 levels in ANH. CXCL12 activated CXCR4, which triggered AKT and NFκB signalling pathways. Consequently, AKT signalling induced the upregulation of collagen type I. MLiM were significantly encircled by a shield of collagen, whereas other liver metastases showed reduced levels of collagen. Of all the liver metastasis analyzed, the presence of collagen in melanoma liver metastasis was associated with a reduction in tumour‐infiltrating lymphocytes.

**Conclusions:**

MLiM modified ANH to increase collagen production and created a physical barrier. The collagen barrier was associated with a reduction of immune cell infiltration which could potentially deter MLiM immune surveillance and treatment responses.

**Highlights:**

- Spatial analyses of melanoma liver metastasis show that adjacent normal hepatocytes have increased collagen‐type I levels.
- Melanoma liver metastases tumour cells secrete enhanced levels of TNF‐α to stimulate CXCR4/CXCL12 upregulation in adjacent normal hepatocytes.
- Activation of CXCR4 promotes AKT and NF‐κB signalling pathways to promote collagen‐type I secretion in adjacent normal hepatocytes.
- Elevated collagen levels were associated with reduced tumour‐infiltrating lymphocytes

## Graphical abstract

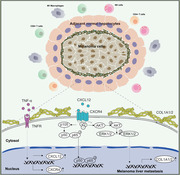

## 1 INTRODUCTION

Cutaneous melanoma (CM) is an aggressive cancer that frequently develops metastasis into different organ sites.^1^ Melanoma liver metastases (MLiM) are one of the most frequent distant metastases in patients with CM whereby prognosis is often poor, with a median survival that ranges from 3 to 6 months once diagnosed.^2^, ^3^, ^4^ The advent of immune checkpoint inhibitors (ICIs) and targeted therapies have improved the prognosis of patients with metastatic melanoma (MM); however, the standard of care treatments are less effective in MLiM than most other organ sites of metastases due to immune resistance.^5^, ^6^, ^7^

Several studies have shown that metastases are supported and progress by their interactions with the surrounding tumour microenvironment (TME) such as the adjacent normal tissue.^8^ In particular, the hepatic microenvironment plays a critical role in regulating the attachment of the tumour cells to specific microcirculatory areas.^8^ MLiM develop various complex mechanisms of interaction with the liver TME that are implicated in promoting tumour metastasis and immunotherapy resistance.^9^, ^10^, ^11^ Therefore, we hypothesized that the adjacent normal hepatocytes (ANH) located in areas near MLiM cells have a different transcriptomic profile compared with distal normal hepatocytes (DNH) areas due to hostile inflammatory TME generated by MLiM.^12^, ^13^, ^14^ Few studies have focused on ANH in hepatocellular carcinoma and colorectal cancer liver metastasis (CLiM),^15^, ^16^ but the understanding of the potential intercellular crosstalk between ANH and MLiM cells remains unexplored. However, due to the limited number of patients with MLiM who undergo surgery, there is a gap in the field.

To identify potential intercellular interactions, the gene expression profiles of ANH, DNH, as well as melanoma tumour cells, were characterized in treatment naïve MLiM using NanoString GeoMx digital spatial profiling (NGDSP, Nanostring Technologies, Inc.) combined with Cancer Transcriptome Atlas (CTA) assay.^17^ Spatial transcriptomic analyses and functional assays demonstrated that MLiM released TNF‐α which stimulates the transcription of *CXCR4/CXCL12* in ANH. CXCR4/CXCL12 complex activated AKT and NFκB signalling pathways. Consequently, *COL1A1/2* mRNA levels significantly increased, and higher levels of collagen were released by ANH. Enhanced collagen deposition in human MLiM created a shield around the MLiM and a physical barrier that may contribute to the reduction of immune cell infiltration including CD8^+^ T cells, CD4^+^ T cells, macrophages, or NK cells. This collagen shield was predominantly observed in MLiM, but to a lesser extent in the most frequently spread liver metastasis such as CLiM and pancreatic liver metastasis (PLiM).

## 2 RESULTS

Spatial transcriptomic analysis of melanoma liver metastasis: upregulated gene signatures in adjacent normal hepatocytes

**FIGURE 1:**
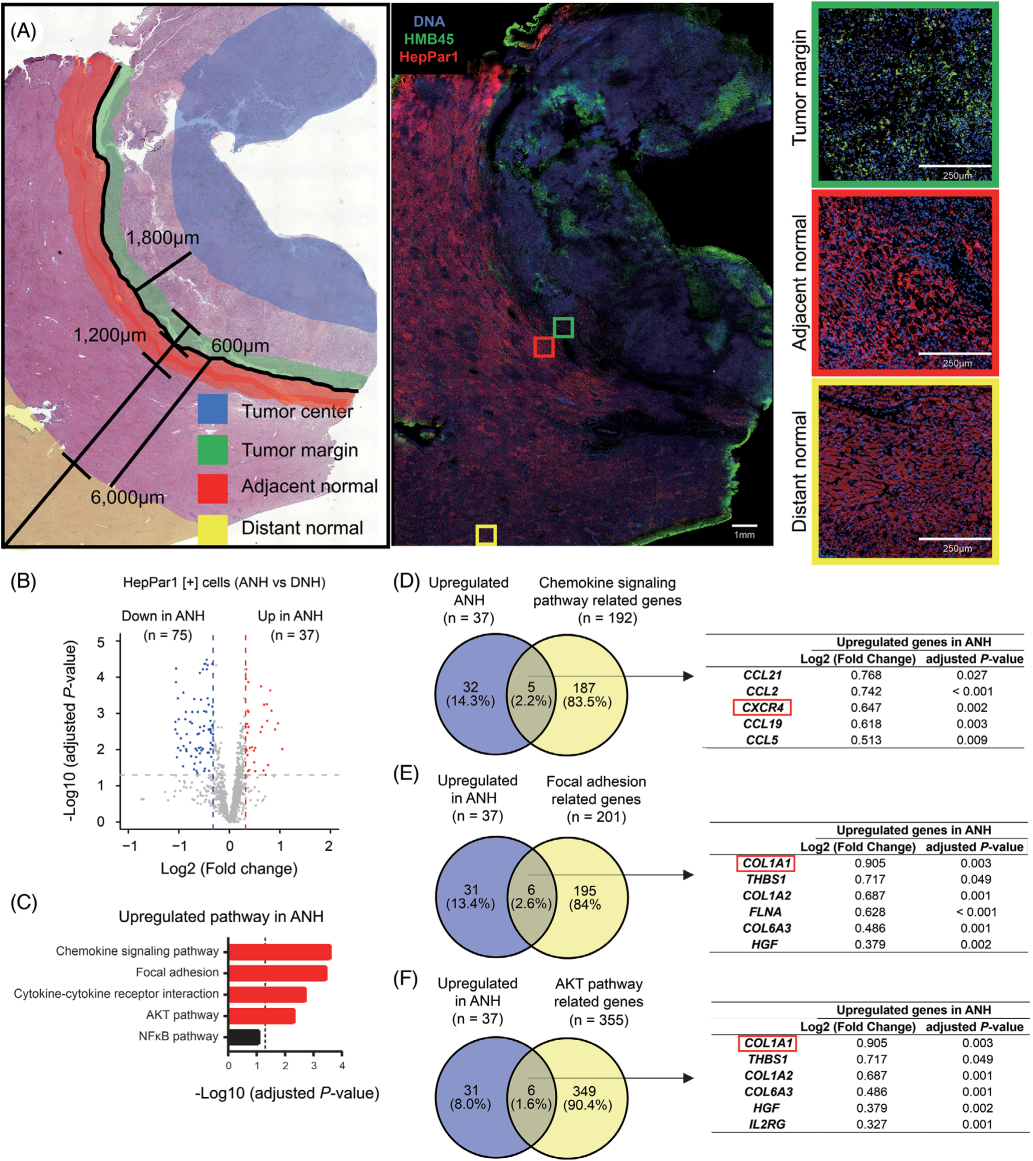
Spatial transcriptomic analysis of the TME of MLiM using NanoString GeoMx DSP (NGDSP). (A) The schema of the study design includes tumour margin (TM), tumour center (TC), adjacent normal hepatocytes (ANH), and distant normal hepatocytes (DNH) tissue areas. (B) Volcano plot showing the DEGs in HepPar1[+] comparing ANH and DNH obtained by NGDSP analysis. Of 112 DEGs, 37 were upregulated (red dots) and 75 were downregulated (blue dots) in ANH. (C) Bar graph showing the upregulated pathways in HepPar1[+] in ANH by KEGG pathway analysis. (D) Venn diagram showing the overlapping genes among the upregulated ANH and chemokine signalling pathway‐related genes. The table shows the genes common to the two groups. (E) Venn diagram showing the overlapping genes among the upregulated in ANH and focal adhesion‐related genes. The table shows the genes common to the two groups. (F) Venn diagram showing the overlapping genes among the upregulated ANH and AKT signalling pathway‐related genes. The table shows the genes common to the two groups.

Spatial transcriptomic analysis using the NGDSP was performed to explore the potential cell–cell interactions linking normal hepatocytes and melanoma cells of MLiM. Using NGDSP, 43 areas of illumination (AOI)s were selected from four patients with untreated MLiM at the time of surgery. AOI's selection was based on known morphology biomarkers: SYTO13 (nucleus), HepPar1 (hepatocytes), and Human Melanoma Black 45 (HMB45; melanoma cells). The AOIs were selected to analyze the AN, DN, tumour margin, and tumour centre areas based on the histopathology of the MLiM tissues (Figure 1A). The specificity of all the AOIs collected was confirmed by assessing the mRNA levels of melanoma markers (*S100B* and *MLANA*) or hepatocyte markers (*ARG1* and *HAMP*). *S100B* and *MLANA* were significantly upregulated in melanoma cells AOIs (Figure S1A,B), and *ARG1* and *HAMP* were significantly upregulated in ANH and DNH AOIs (Figure S1C,D), confirming the specificity of AOIs selection using the above‐mentioned morphological markers. Therefore, from this point on and for simplification, the AOIs including HepPar1[+] cells in AN or DN will be referred to as ANH or DNH respectively. Similarly, the AOIs including HMB45[+] cells in tumour margin and tumour centre areas will be referred to as melanoma cells.

The 14 AOIs from ANH and 9 AOIs from DNH were compared with examine the differential expressed genes (DEGs). Of the 1811 genes assessed in the NGDSP CTA, 37 genes were upregulated, and 75 genes were downregulated in ANH compared with DNH (Figure 1B; Table S5). KEGG pathway analysis using the 37 upregulated genes showed that the chemokine signalling pathway, focal adhesion, cytokine‐cytokine receptor interaction, and AKT signalling pathway were significantly enriched in ANH (Figure 1C). Five genes (*CCL21*, *CCL2*, *CXCR4*, *CCL19*, and *CCL5*) of the chemokine signalling pathway were commonly upregulated in ANH (Figure 1D). Similarly, six genes (*COL1A1*, *THBS1*, *COL1A2*, *FLNA*, *COL6A3*, and *HGF*) of the focal adhesion as well as six genes (*COL1A1*, *THBS1*, *COL1A2*, *COL6A3*, *HGF*, and *IL2RG*) of the AKT signalling pathway were commonly upregulated in ANH (Figure 1E,F). Among the significantly upregulated genes in ANH, we focused on CXCR4 and COL1A1/2.

Previously, our group showed that high CXCR4 levels in MLiM tend to be associated with a worse prognosis.^18^ A recent study showed that liver metastases with high CXCR4 levels have a better response to CXCR4 antagonist combinational therapy with ICI.^19^, ^20^ COL1A1 is significantly elevated in ANH and associated with immunosuppression.^21^ Consistently, *CXCR4* and *COL1A1* mRNA levels were significantly correlated at mRNA expression in ANH (Figure S1E–I).

Seven AOIs from the tumour margin and 13 AOIs from the tumour centre were analyzed to identify DEGs in melanoma cells. However, no significant changes were found at the transcriptomic levels in melanoma cells obtained from the tumour margin and tumour centre of MLiM (Figure S2A). Therefore, the 20 AOIs from melanoma cells obtained from the tumour margin and tumour centre were combined and compared with the 14 AOIs from ANH to identify DEGs. While 139 genes were upregulated, 230 genes were downregulated in melanoma cells (Figure S2B; Table S6). KEGG pathway analysis using the 139 upregulated genes showed that cell cycle, AKT signalling pathway, MAPK signalling pathway, JAK‐STAT signalling pathway, TNF signalling pathway, and cytokine–cytokine receptor interaction were significantly upregulated in melanoma cells (Figure S2C). Five genes (*CXCL10*, *TNF*, *PIK3CD*, *PIK3R2*, and *CXCL1*) of the TNF signalling pathway and seven genes (*CXCL9*, *GDF15*, *BMP7*, *TNFRSF15*, *CXCL10*, *TNF*, and *CXCL1*) of the cytokine–cytokine receptor pathway were commonly upregulated in melanoma cells (Figure S2D,E). Among the commonly upregulated genes in tumour areas, the focus was on TNF as it is highly expressed in melanoma cells.^22^ TNF has recently received attention as a target in combinational therapies with ICIs for MM treatment.^23^, ^24^ In conclusion, ANH from MLiM showed an upregulation in *CXCR4* and *COL1A1/2*, while melanoma cells of MLiM showed a significant upregulation in TNF.

**FIGURE 2:**
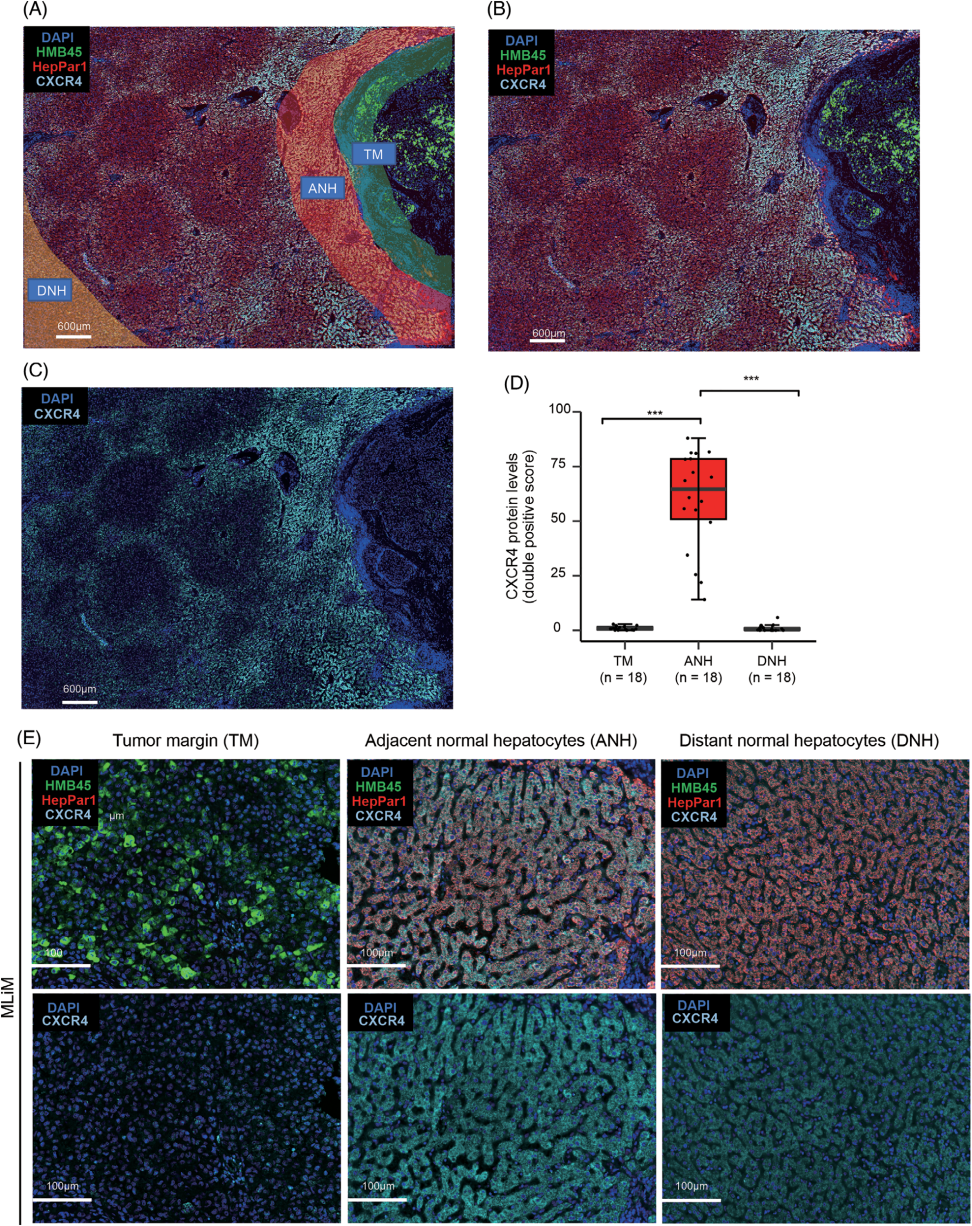
Representative mIF images of CXCR4 staining patterns of MLiM. (A) Representative image of the histological areas (tumour margin (TM), adjacent normal hepatocytes (ANH), and distant normal hepatocytes (DNH)) in MLiM FFPE tissues. (B, C) Representative mIF images of MLiM of the merge image (DAPI‐HMB45‐HepPar1‐CXCR4, B) and CXCR4 (DAPI‐CXCR4, C) from FFPE tissue samples that were stained using Opal mIF assay. DAPI (blue); HMB45 (green); HepPar1 (red); CXCR4 (cyan). Scale bar = 600 µm. (D) Quantification of CXCR4 protein levels in HepPar1[+] cells in ANH and DNH, and in HMB45[+] cells in TM. (E) Representative image of MLiM in TM, ANH, and DNH. Scale bar = 100 µm. Data represents the mean ± SD. ***p < .001.

To validate the *CXCR4* and *COL1A1* mRNA upregulation observed in spatial transcriptomic analysis, the CXCR4 protein levels and distribution were evaluated by multiplex immunofluorescence (mIF). Significantly higher CXCR4 protein levels were uniformly distributed in HepPar1[+] cells (hepatocytes) in the ANH area compared with DNH or HMB45[+] (melanoma cells) tumour areas (Figure 2A–E; Figure S3A). Of note, similar results were observed in tissue biopsies obtained from MLiM (Figure S4A–D).

Previously, our group showed that KPC1 is a key molecule regulating the amount and balance of p50–p50 dimers.^25^ Downregulation of the ubiquitin‐E3 ligase KPC1 reduces p105 processing into p50 levels. Downregulation of p50 influences the balance toward p50–p65 dimers formation and the NFκB signalling pathway activation, which plays a significant role in MM progression.^25^ In addition, CXCR4 activates the AKT and NFκB signalling pathway in melanoma cells to promote cell survival and proliferation.^20^, ^26^ However, in normal hepatocytes, the activation of the NFκB signalling pathway promotes inflammation.^27^ Furthermore, there is evidence suggesting that NFκB activation increases collagen expression in normal hepatocytes and tumour cells.^28^, ^29^, ^30^, ^31^, ^32^

To determine any potential downstream pathways connecting CXCR4 and COL1A1, p50 and p65 protein levels were analyzed by mIF. Significantly higher p50 and p65 protein levels were observed in ANH compared with DNH or melanoma cells (Figures S5A–D and S6A). In summary, CXCR4 protein levels were significantly upregulated in ANH, confirming the spatial transcriptomic results. Furthermore, p50 and p65 protein levels were significantly upregulated in ANH, suggesting potential interactions between ANH and melanoma cells of MLiM.

### 2.1 MLiM cells supernatant activated CXCR4, AKT, and NFκB signalling pathways and increased collagen secretion in hepatocytes

**FIGURE 3:**
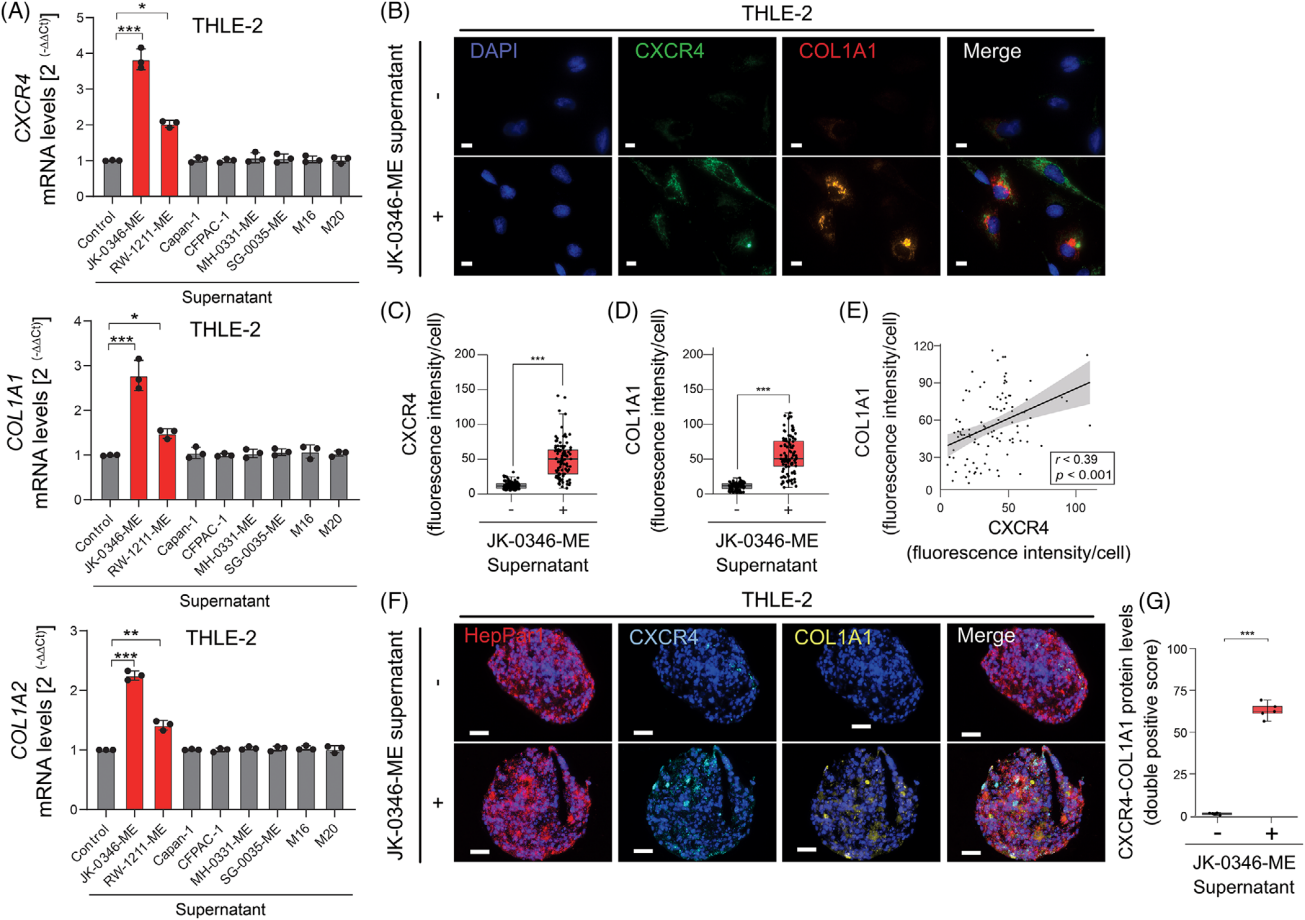
CXCR4 and COL1A1 expression of THLE‐2 cells after treatment of MLiM and PLiM supernatants. (A) Quantification of *CXCR4* and *COL1A1/A2* mRNA levels in THLE‐2 cells in control conditions and after incubation with MLiM (JK‐0346‐ME or RW‐1211‐ME) or PLiM cell lines (Capan1 or CFPAC1) supernatants. (B) Representative images of CXCR4 and COL1A1 expression of THLE‐2 cells after incubation with MLiM cell line (JK‐0346‐ME) supernatant. Scale bar = 10 µm. (C) Quantification of CXCR4 fluorescence intensity using Qupath software. (D) Quantification of COL1A1 fluorescence intensity using Qupath software. (E) Correlation between the intensity of CXCR4 and COL1A1 after MLiM supernatant incubation. (F) Representative mIF image of normal hepatocyte spheroids incubated with or without MLiM cell line (JK‐0346‐ME) supernatants. Spheroids were stained using Opal mIF assay. DAPI (blue); HepPar1 (red); CXCR4 (cyan); COL1A1 (yellow). Scale bar = 50 µm. G Quantification of the percentage of cells with CXCR4 and COL1A1 co‐expression using a double positive score. Data represents the mean ± SD. NS: not significant, **p < .01, ***p < .001. The correlation was determined by Spearman's correlation test E.

PLiM and CLiM represent the largest population of all patients diagnosed with liver metastases^33^; therefore, available PLiM cell lines were selected as control groups for MLiM. Additionally, melanoma lymph node metastasis (MLNM) and melanoma brain metastasis (MBM) represent two of the most common sites of MM. Therefore, MLNM and MBM cell lines were assessed as controls for the specificity of the changes observed in MLiM. To functionally validate the transcriptomic results obtained from NGDSP, the cell culture supernatants of MLiM (JK‐0346‐ME and RW‐1211‐ME), PLiM (Capan‐1 and CFPAC1), MLNM (MH‐0331‐ME and SG‐0035‐ME), or MBM (M16 and M20) cell lines were collected and administered to the culture medium of THLE‐2 cell line. RT‐qPCR analysis demonstrated that *COL1A1/2* and *CXCR4* mRNA levels were significantly enhanced in THLE‐2 cells when MLiM supernatant was administered (Figure 3A). *COL1A1/2* and *CXCR4* mRNA levels did not change significantly when PLiM, MLNM, or MBM cell line supernatants were administered to THLE‐2 cells (Figure 3A). Of note, MLiM cell lines produced low levels of *COL1A1/2* compared with THLE‐2 in resting or stimulated conditions (Figure S7A,B). Then, we analyzed 3D spheroids in culture generated from the THLE‐2 cell line incubated with and without MLiM supernatant. The administration of MLiM supernatant increased the expression of CXCR4 and COL1A1 in THLE‐2‐derived spheroids (Figure 3F,G). To summarize, MLiM cell line supernatant increased CXCR4 and COL1A1/2 levels in normal hepatocytes. The experiments demonstrated the specificity of MLiM cell supernatant to induce CXCR4 and COL1A1/2 compared with metastatic cell lines of different cancer types as well as different melanoma organ metastasis.

**FIGURE 4:**
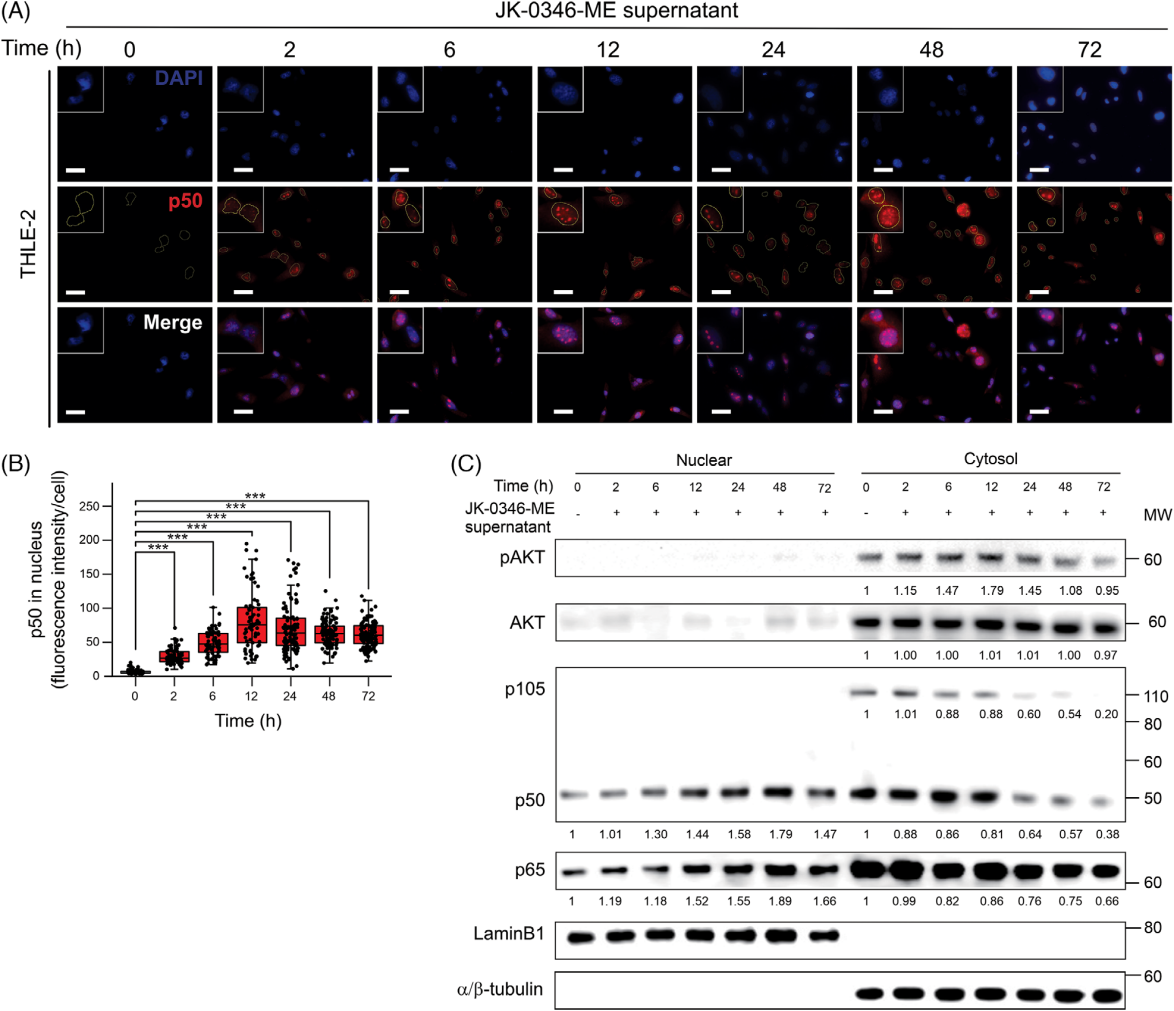
Activation of NFκB and AKT signalling pathway in THLE‐2 cells after treatment with MLiM cells supernatant. (A) Representative images showing the p50 protein levels in THLE‐2 cells after the incubation with MLiM cell line (JK‐0346‐ME) supernatant. Scale bar = 50 µm. (B) Quantification of p50 (B) fluorescence intensity in the nucleus of THLE‐2 cell lines using Qupath software. (C) Western blot of p‐AKT, AKT, p105, p50, p65 in the nuclear and cytosol fractions of THLE‐2 cells after treatment of MLiM cell line (JK‐0346‐ME) supernatant. Lamin B1 was used as the loading control for the nuclear fractions and α/β‐tubulin was used as the loading control for the cytosol fractions. Data represents the mean ± SD. *** *p* < .001.

To understand the relation between CXCR4 and COL1A1 expression, we further focused on the AKT and NFκB signalling pathways. The AKT signalling pathway activation was determined by assessing the phosphorylated levels of AKT (p‐AKT), while the NFκB pathway was assessed by measuring the levels of p105 and p50, followed by p50 and p65 translocation into the nucleus.^25^ The increase in p‐AKT and p50 levels only occurred when adding MLiM supernatant, but not when adding PLiM, MLNM, or MBM supernatant (Figure S8A–I). Significantly higher p50 protein levels were observed in the nucleus compartment two hours after MLiM supernatant administration to THLE‐2 cells (Figure 4A–B). In western blot analysis, AKT activation significantly increased in cytoplasm fractions after MLiM supernatant administration to THLE‐2 cells (Figure 4C). Supporting our observations, the protein levels of p50 and p65 increased in the nuclear fraction, while the levels of p50 and p105 decreased in the cytosolic fraction (Figure 4C), suggesting the NFκB signalling pathway activation. In conclusion, MLiM cell lines supernatant activated AKT and NFκB signalling pathways in THLE‐2 cells, while this effect was not observed when using other liver metastatic cell lines or different melanoma organ metastasis‐derived cell lines.

### 2.2 CXCR4 knockdown prevented AKT and NFκB signalling pathway activation and reduced COL1A1 expression in hepatocyte cell line

**FIGURE 5:**
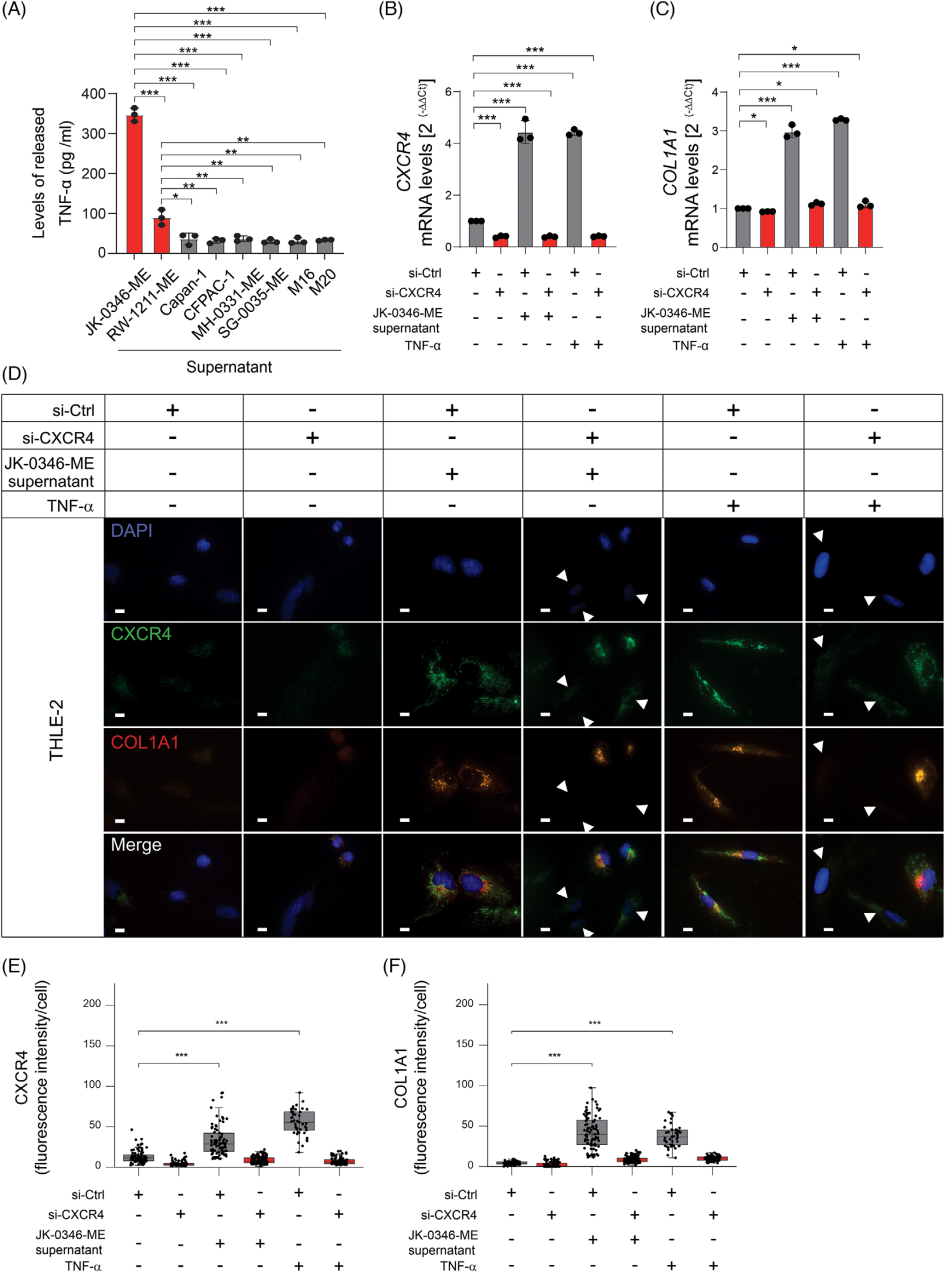
CXCR4 knockdown decreased COL1A1 levels in THLE‐2 cells. (A) Quantification of released TNF‐α levels from MLiM (JK‐0346‐ME, RW‐1211‐ME), PLiM (Capan‐1, CFPAC‐1), MLNM (MH‐0331‐ME and SG‐0035‐ME), or MBM (M16 and M20) cell lines. (B) Quantification of *CXCR4* mRNA levels in THLE‐2 control (si‐Ctrl) or CXCR4 knockdown (si‐CXCR4) cells after incubation with MLiM supernatant or TNF‐α treatment. (C) Quantification of *COL1A1* mRNA levels in THLE‐2 si‐Ctrl or si‐CXCR4 cells after incubation with MLiM supernatant or TNF‐α treatment. D Representative images of CXCR4 and COL1A1 protein levels in THLE‐2 si‐Ctrl or si‐CXCR4 cells after incubation with MLiM supernatant or TNF‐α treatment. Scale bar = 10 µm. (E) Quantification of CXCR4 fluorescence intensity using Qupath software. (F) Quantification of COL1A1 fluorescence intensity using Qupath software. Data represents the mean ± SD. NS, not significant, *p < .05, **p < .01, ***p < .001.

Based on the AKT and NFκB signalling pathways activation in THLE‐2 cells and the observation that the TNF−signalling pathway was upregulated in spatial transcriptomic analysis, we hypothesized that the MLiM secrete high levels of TNF‐α. Enzyme‐linked immunoassay (ELISA) assays demonstrated that MLiM cell lines released significantly higher levels of TNF‐α than PLiM, MLNM, or MBM cell lines (Figure 5A). Since, TNF‐α promotes the transcription of CXCR4, but not CXCR4 activation, the THLE‐2 cell line was treated with TNF‐α to determine the levels of CXCL12. THLE‐2 cell line showed increased CXCL12 mRNA levels after MLiM supernatant or TNF‐α administration (Figure S7C). Supporting these findings, mIF staining showed significantly higher protein CXCL12 levels in ANH compared with DNH from MLiM (Figure S9A–E).

To demonstrate that CXCL12‐CXCR4 activation promotes COL1A1 expression, THLE‐2 cell lines with CXCR4 knockdown were treated with MLiM cell culture supernatant or TNF‐α. Under these conditions, COL1A1/2 did not significantly increase (Figure 5B–F; Figure S7D). Independent of CXCR4 presence, CXCL12 mRNA levels significantly increased upon MLiM cell culture supernatant or TNF‐α treatment, suggesting that CXCL12 is upstream of CXCR4 (Figure S7E). The results suggested that TNF‐α depends on CXCR4 to increase COL1A1/2 mRNA and protein levels in normal hepatocytes.

**FIGURE 6:**
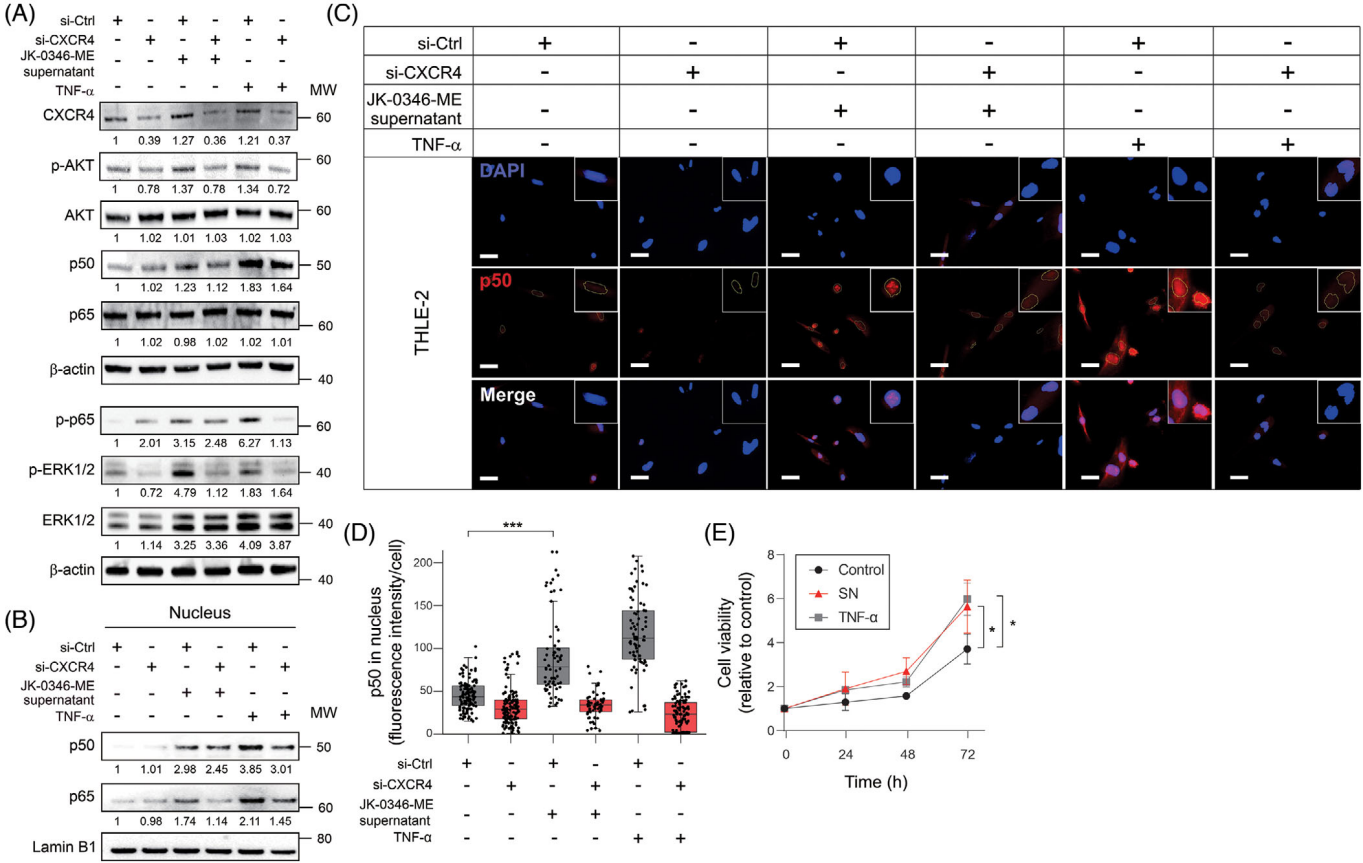
CXCR4 knockdown decreased AKT and NFκB pathways activation. (A) Western blot of p‐AKT, AKT, p50, p65, p‐p65, p‐ERK1/2, ERK1/2 in THLE‐2 control (si‐Ctrl) or CXCR4 knockdown (si‐CXCR4) cells after incubation with MLiM supernatant or TNF‐α treatment. (B) Western blot of p50 and p65 in the nuclear fraction of THLE‐2 after MLiM cells supernatant or TNF‐α treatment with CXCR4 knockdown or not. Lamin B1 was used at the loading control for the nuclear fractions. (C) Representative images showing p50 levels in THLE‐2 si‐Ctrl or si‐CXCR4 cells after incubation with MLiM supernatant or TNF‐α treatment. Scale bar = 10 µm. (D) Quantification of p50 fluorescence intensity in the nucleus using Qupath software. (E) Cell viability assay for THLE‐2 after incubation with MLiM cells supernatant or TNF‐α treatment. Data represents the mean ± SD. *p < .05, ***p < .001.

To validate whether AKT and NFκB signalling pathways activation were downstream mediators upon CXCR4 activation, THLE‐2 cell lines with CXCR4 knockdown were treated with MLiM supernatant or TNF‐α, and then assessed by western blot. CXCR4 knockdown significantly decreased p‐AKT and pERK1/2 levels in untreated conditions (Figure 6A). In THLE‐2 cells with CXCR4 knockdown, the addition of MLiM supernatant or TNF‐α administration—as positive control—did not increase p‐AKT, pERK1/2, or p‐p65 levels (Figure 6A). Next, the p50 and p65 translocation into the nuclear fraction were assessed to confirm the activation of the NFκB signalling pathway. Of note, the levels of p50 and p65 significantly decreased in the nuclear fraction of THLE‐2 cells with CXCR4 knockdown after MLiM supernatant or TNF‐α administration (Figure 6B). Comparable results were observed for p50 in IF assays (Figure 6C,D). Consistent with the AKT signalling pathway activation, the treatment of THLE‐2 cells with MLiM supernatant or TNF‐α‐enhanced cell proliferation (Figure 6E). In conclusion, normal hepatocytes required CXCR4 presence to mediate AKT and NFκB signalling pathways activation upon MLiM supernatant or TNF‐α administration.

### 2.3 COL1A1 production is controlled by the AKT pathway

**FIGURE 7:**
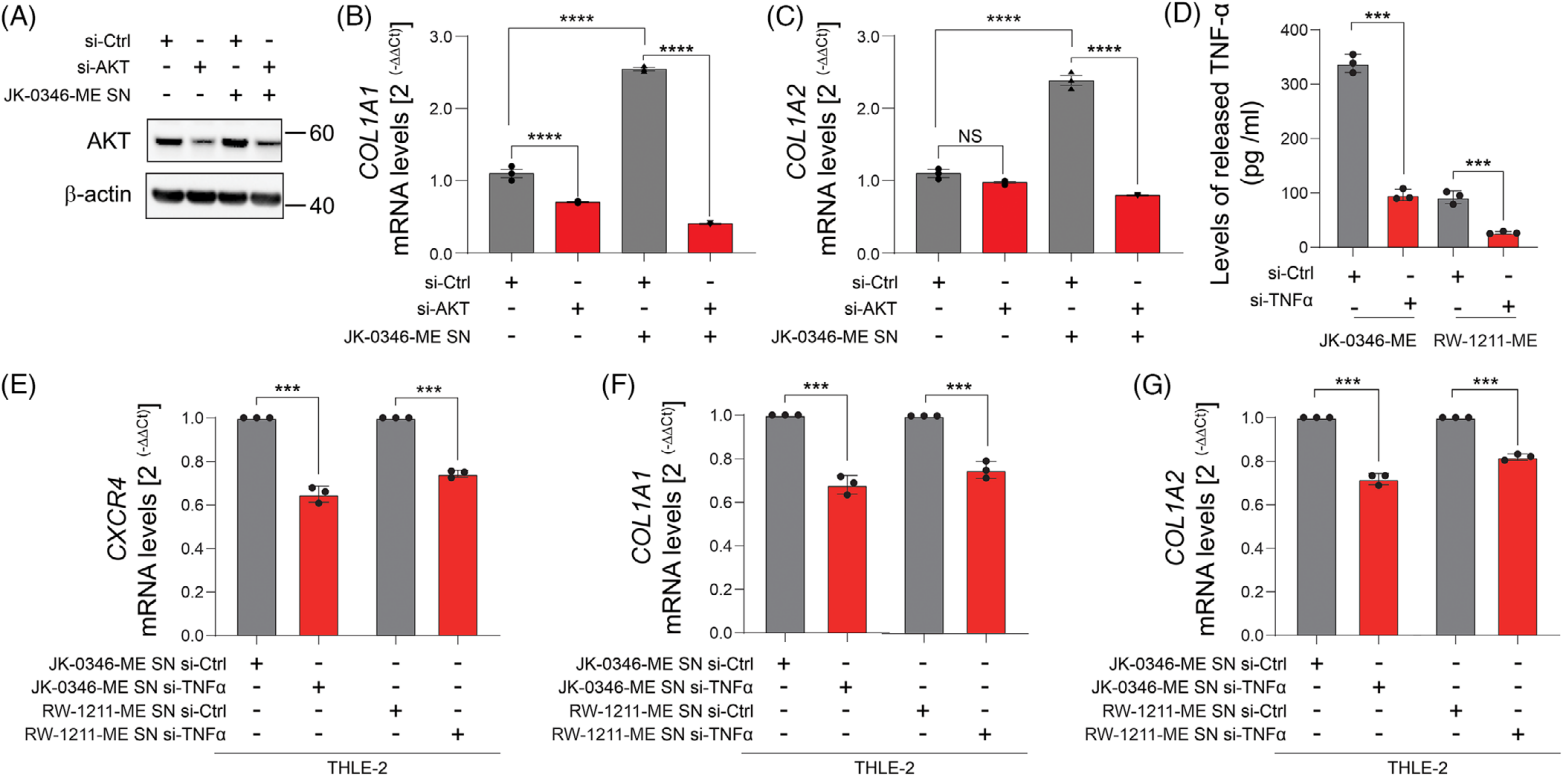
AKT knockdown in THLE‐2 or TNF‐α knockdown in MLiM decreased COL1A1/2 levels in THLE‐2. (A) Western blot for AKT in THLE‐2 control (si‐Ctrl) or AKT knockdown (si‐AKT) incubated with or without JK‐0346‐ME supernatant (SN). (B, C) Quantification of *COL1A1* (B) or *COL1A2* (C) mRNA levels in THLE‐2 control (si‐Ctrl) or AKT knockdown (si‐AKT incubated with or without JK‐0346‐ME supernatant (SN) using RT‐qPCR. (D) Quantification of released TNF‐α levels in MLiM (JK‐0346‐ME, RW‐1211‐ME) control (si‐Ctrl) or TNF‐α knockdown (si‐TNF‐α). RT‐qPCR quantification of *CXCR4* (E), *COL1A1* (F), *COL1A2* (G) mRNA levels in THLE‐2 incubated with supernatants derived from MLiM cell lines (JK‐0346‐ME and RW‐1211‐ME) control (si‐Ctrl), or TNF‐α knockdown (si‐TNF‐α). Data represents the mean ± SD. ***p < .001, ****p < .0001.

To determine the role of Akt pathways in controlling *COL1A1/2* levels, we performed knockdown assays. AKT knockdown was confirmed by western blot (Figure 7A). Of notice, AKT downregulation significantly reduced *COL1A1/2* levels in the THLE‐2 cell line (Figure 7B,C), suggesting that the AKT signalling pathway is critical in controlling collagen production in normal hepatocytes.

In addition, TNF‐α was knockdown in MLiM cell lines and the levels of TNF‐ α were confirmed by ELISA assay (Figure 7D). Then, the supernatants from both MLiM cell lines after TNF‐α knockdown were collected and utilized to incubate with the THLE‐2 cell line. Of note, TNF‐α knockdown in MLiM significantly decreased *CXCR4* as well as *COL1A1/2* mRNA levels in the THLE‐2 cell line (Figure 7E–G), suggesting that TNF‐α mediated the effects of the MLiM supernatants and confirming the previous observations.

### 2.4 COL1A1 production was associated with reduced immune cell tumour infiltration in MLiM, but not PLiM or CLiM

**FIGURE 8:**
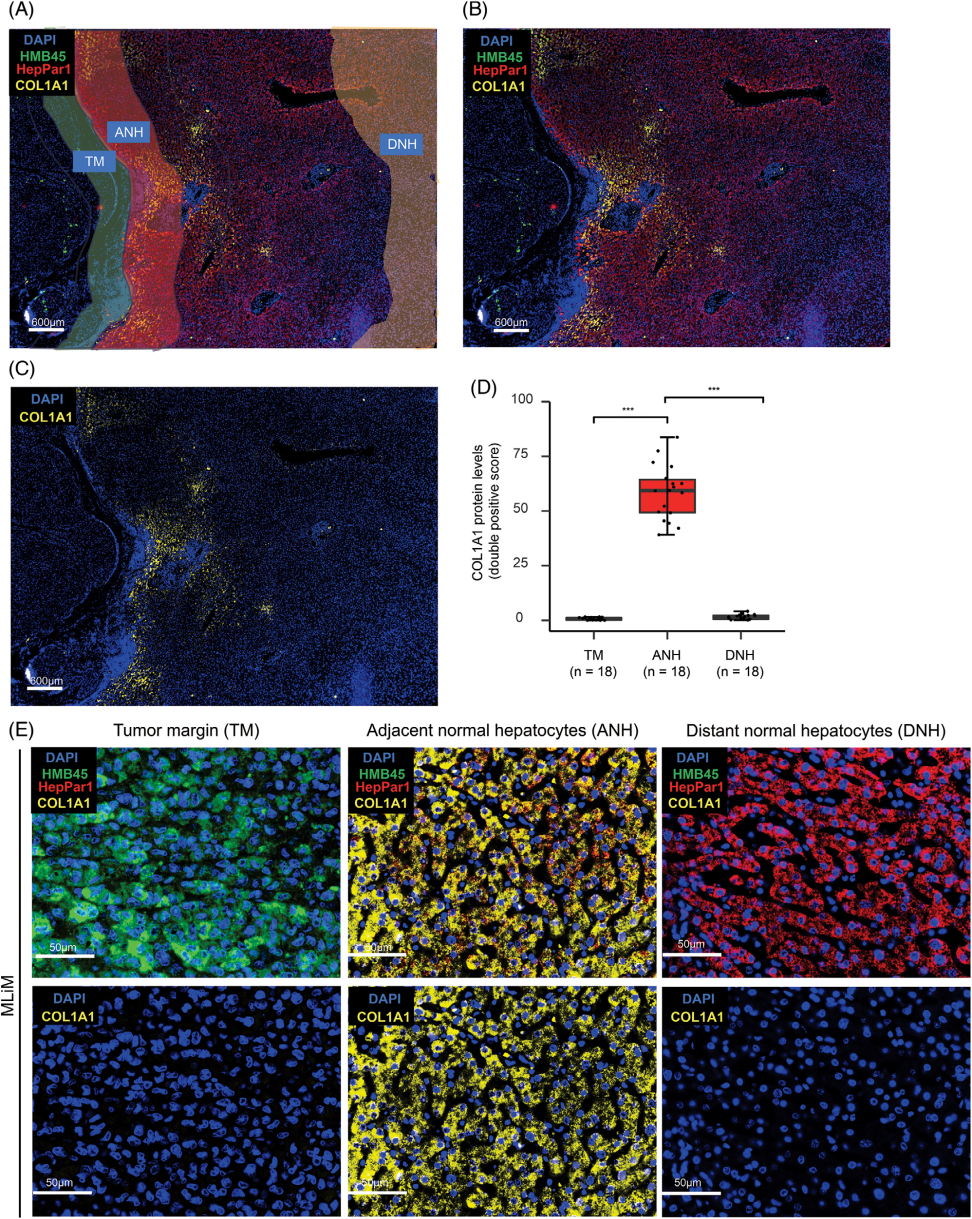
Representative mIF images of COL1A1 staining patterns of MLiM. (A) Representative image of the histological areas (tumour margin [TM], adjacent normal hepatocytes [ANH], and distant normal hepatocytes [DNH]) in MLiM FFPE tissues. (B, C) Representative mIF images of MLiM showing the merged image (DAPI‐HMB45‐HepPar1‐CXCR4, B) and CXCR4 (DAPI‐CXCR4, C) from FFPE tissue samples that were stained using Opal mIF assay. DAPI (blue); HMB45 (green); HepPar1 (red); COL1A1 (yellow). Scale bar = 600 µm. (D) Quantification of COL1A1 protein levels in HepPar1[+] cells in ANH and DNH, and in HMB45[+] cells in TM. (E) Representative images of MLiM in TM, ANH, and DNH. Scale bar = 50 µm. Data represents the mean ± SD. ***p < .001.

To validate our previous observations obtained from spatial transcriptomic analysis, the COL1A1 protein levels and distribution were evaluated by mIF. Significantly higher COL1A1 protein levels were observed in ANH than in DNH or melanoma cells of MLiM (Figure 8A–E; Figure S10A). Of note, similar results were observed in tissue biopsies obtained from MLiM (Figure S11A–D).

Then, we compared the levels of collagen produced by MLiM as well as CLiM and PLiM. The protein levels of COL1A1 in ANH of CLiM and PLiM were measured by mIF and Masson's Trichrome staining using serial formalin‐fixed‐paraffin‐embedded (FFPE) tissues. Significantly higher COL1A1 protein levels (mIF) or total collagen levels (Masson's Trichrome Stain) were observed in ANH from MLiM compared with ANH from CLiM or PLiM (Figures S12A–F and S13A–C). Next, THLE‐2 cell lines were incubated with MLiM or PLiM cell culture supernatants, suggesting a unique mechanism driven by MLiM supernatant.

Next, we examined the level of tumour‐infiltrating lymphocytes (TILs) in MLiM tissues given the physical barrier created by a large amount of collagen deposition. The proportion of CD8^+^ T cells, CD4^+^ T cells, or NK cells (CD3^–^/CD56^+^) was significantly lower in MLiM than in CLiM or PLiM tumour areas (Figures S14A,B and S15A,B). The results suggested that the COL1A1 upregulation in ANH, secretion, and deposition in MLiM may represent a physical barrier for immune cells to infiltrate MLiM tumours.

## 3 DISCUSSION

In this study, we found that MLiM released TNF‐α to promote the upregulation of CXCL12 and CXCR4 in ANH. The CXCL12/CXCR4 axis triggers the activation of AKT‐ and NFκB signalling pathways. AKT signalling pathway promotes the elevation of COL1A1/2 levels in ANH. Consequently, increased levels of extracellular collagen deposition were associated with a reduction of CD8^+^ T cells, CD4^+^ T cells, macrophages, and NK cells tumour infiltration in MLiM.

Previous studies showed that the CXCL12/CXCR4 axis contributed to the formation of a pre‐metastatic niche for metastatic melanoma cells and metastasis development.^34^, ^35^ The limitation is that these previous studies focused on the CXCL12/CXCR4 function on metastatic melanoma cells but not in the adjacent cells that are part of the TME. This study demonstrated that CXCR4 and CXCL12 are significantly elevated in ANH of the TME of MLiM, suggesting that the ANH may have biological implications in MLiM development/progression. CLiM and PLiM are the most common types of liver metastasis and account for more than half of all diagnosed liver metastases.^33^ Therefore, CLiM and PLiM were included as a control group for comparison. In mIF analysis, CXCR4 and CXCL12 protein levels were significantly elevated in ANH of MLiM, but not in ANH of CLiM or PLiM tissue samples, suggesting that CXCR4 upregulation was specific to MLiM.

Spatial transcriptomic analysis demonstrated an upregulation of the TNF signalling pathway in melanoma cells of MLiM. A previous study showed that aggressive melanoma cells release increasing levels of TNF‐α.^22^ The results demonstrated that normal hepatocytes exposed to supernatant collected from the MLiM cell line increased the expression of CXCR4 and COL1A1. ELISA assay confirms that MLiM, but not other types of melanoma organ‐metastasis such as MLNM or MBM, secretes enhanced levels of TNF‐α. Also, PLiM‐derived cell lines did not release significantly higher levels of TNF‐α compared with MLiM cell lines, suggesting that this effect is highly specific to MLiM.

Previous reports showed that CXCR4 activates the AKT signalling pathway.^26^, ^36^, ^37^ In addition, the NFκB signalling pathway is downstream of CXCR4/CXCL12,^26^, ^37^ and the NFκB signalling pathway can activate the AKT signalling pathway.^27^ Also, NFκB activation is known to increase collagen expression in hepatocytes.^31^, ^32^ A previous study showed that treatment with tormentic acid, which has a hepatoprotective effect, suppressed collagen expression in hepatocytes by inhibiting AKT and NFκB signalling pathways.^31^ Another study showed that the knockout of Kindlin‐2, which is a key component of focal adhesion, activated the NFκB signalling pathway and increased the expression of collagen in hepatocytes.^32^ Our results showed that the supernatant obtained from MLiM cell lines and administered to THLE‐2 increased the phosphorylation levels of AKT. Furthermore, p50 and p65 levels were significantly elevated in the nucleus of normal hepatocytes, suggesting NFκB pathway activation. However, only AKT was responsible for the upregulation of *COL1A1/2* mRNA levels.

Previous studies showed that collagen expression led to ICI treatment resistance in metastatic melanoma.^21^, ^38^ However, these studies focused on the collagen released by tumour cells and not on ANH or other stromal cells. COL1A1 levels were significantly elevated in ANH of MLiM but not in other types of liver metastasis (CLiM and PLiM). Of note, the contribution of collagen production by melanoma cells derived from MLiM is minimal compared with that released by normal hepatocyte‐derived cell lines. It has also been reported that the physical barrier provided by the extracellular matrix prevents T cells from penetrating the tumour sites, resulting in immunosuppression.^39^, ^40^ Therefore, the elevated collagen production in ANH of MLiM suggests that collagen forms a physical barrier to prevent immune cell infiltration in the MLiM tumour.

Previous studies showed that aggressive melanoma cells release TNF‐α, which elicits melanoma dedifferentiation, promoting immune escape and melanoma relapse.^22^, ^41^ TNF‐α also enhances the expression of programmed cell death ligand 1 (PD‐L1) in tumour cells including melanoma.^42^, ^43^ and TNF‐α blockade leads to an increased content of CD8^+^ TILs, which is a predictive factor of ICIs’ treatment response in MM patients.^23^, ^44^ Furthermore, it has been reported that TILs’ presence is significantly less common in MLiM and MBM than in MLNM or melanoma lung metastases.^4^, ^7^, ^45^ These studies show that TNF‐α elevated PD‐L1 expression and suppressed TILs have led to the combination of ICIs and TNF inhibitors in preclinical and clinical studies.^23^, ^24^ Furthermore, a combination of ICIs and CXCR4 inhibitors in melanoma was shown to increase the expression of CD8^+^ TILs and significantly reduce tumour growth.^19^, ^20^ However, the detailed rationale of these combinational therapies containing anti‐TNF‐α or anti‐CXCR4 with ICIs remains unclear. The main concern is that all these studies have focused on the events occurring within the tumour, ignoring the importance of the adjacent normal TME. Our study helps to characterize ANH from MLiM, which may help elucidate the molecular mechanisms of efficacy of these combinational therapies, but also unravel some mechanisms of resistance.

In conclusion, our study showed that TNF‐α released by melanoma cells caused molecular biological changes in the ANH of MLiM. The mechanism proposed explains how the TME and more specifically the ANH are stimulated by MLiM to secrete increased levels of collagen via AKT and NFκB signalling pathways activation. Therefore, increasing collagen production creates a barrier that may reduce immune cells such as CD8^+^ T cells, CD4^+^ T cells, and NK cells (CD3^−^ and CD56^+^) infiltration and tumour immune surveillance. These studies indicate that the adjacent TME of MLiM plays a significant role in liver metastasis progression and resistance.

## 4 MATERIALS AND METHODS

### 4.1 Ethics approval

The study was conducted following the Declaration of Helsinki. Human samples and clinical information for this study were obtained according to the protocol guidelines approved by the SJHC/SJCI Joint Institutional Review Board (IRB) and Western IRB: MORDRTPCR‐0995.

### 4.2 Patient selection

The study was conducted following the Declaration of Helsinki. The study was approved by the Ethics Committee at Saint John's Cancer Institute (SJCI): MORD‐RTPCR‐0995. The cohort consisted of patients who underwent surgery and were pathologically diagnosed with MLiM (*n* = 11), CLiM (*n* = 6), and PLiM (*n* = 3) at Providence Saint John's Health Center (SJHC). The quality of all FFPE sections was evaluated using hematoxylin and eosin staining. All FFPE sections included in the study were evaluated by a gastrointestinal specialist pathologist at the Surgery Pathology Department, SJHC. Tissues were excluded if >25% necrotic areas or fibrosis were observed. The clinical and pathological information data—including age at diagnosis, gender, race, and preoperative treatment—of the surgical resected specimens collected from each patient is described in Table S1. The analyses performed in each specimen are also described in Table S1. A cohort of seven tissue biopsies for patients diagnosed with MLiM were included in the study.

### 4.3 NanoString GeoMx DSP analysis

Tissue preparation and procedure were performed according to the vendor's protocol with some modifications as previously described.^46^ The antibodies (Abs) utilized are summarized in Table S2. Additionally, the regions of interest (ROI)s were segmented into hepatocyte paraffin 1 (HepPar1, hepatocytes) positive [+] cells or HMB45 (melanoma cells) positive [+] cells to define the specific AOIs. A total of 43 AOIs were carefully reviewed for segments with sequencing saturation <25%, nuclei fewer than 80, negative probe counts <3, or a surface area of <12 000 squared microns. All 43 AOIs were included for further analysis following the NanoString user's manual recommendations (Figure 1A). Background correction and scaling were performed using geometric means and normalization was performed using Q3 averages of housekeeping genes. Genes with expression levels at or lower than the limit of quantification in at least 5% of segments were filtered out. A total of 1811 genes passed quality control and were analyzed.

A total of seven ROIs with tumour margins and 13 ROIs with tumour centres were selected, respectively. Tumour margin was defined as the areas on the border of malignant tumour cells close to normal tissue based on pathological features. Tumour centre was defined as central tumour tissue. In addition, 14 ROIs were selected at ANH. The ANH areas were defined as an area within 1200 µm from the TM. Furthermore, nine ROIs were selected in the DNH areas. DNH was defined as an area at least 6 mm away from tumour margin (Figure 1A).

### 4.4 Multiplex immunofluorescence staining

mIF was performed using the Opal 7‐colour manual IHC Kit (NEL 811001KT, Akoya Biosciences), which relies on individual tyramide signal amplification‐conjugated fluorophores to detect various targets. mIF staining was performed as previously described.^46^ except for Abs utilized, the selected panels, and the corresponding fluorophores that are summarized in Table S3. The information for patients included in the staining is shown in Table S1. MIF‐stained slides were imaged using the Mantra Multispectral Imaging System (Akoya Biosciences) as previously described.^46^ For the scoring of targeted protein levels, a double positive score was calculated using the InForm software (Akoya Biosciences) according to the manufacturer's instructions and as previously described.^46^, ^47^ The optical signal threshold to classify the double positive score was set to 1.330 for HepPar1, 1.330 for HMB45, 0.600 for PanCK, 1.250 for CXCR4, 1.260 for CXCL12, 1.500 for COL1A1, 1.330 for p50, and 1.280 for p65, respectively. For double positive scores, the protein‐positive areas were automatically segmented, and nucleus/cytoplasm compartments were distinguished automatically by detecting the intensity of nucleus staining. For validation analysis, all the markers mentioned were evaluated in MLiM (*n* = 6), CLiM (*n* = 6), and PLiM (*n* = 3). For each tissue analyzed, optimal three to six photographs were captured at 20× magnification.

### 4.5 Collagen staining

Collagen staining was performed using Masson's Trichrome Stain Kit (Polysciences). Staining was performed following the manufacturers’ instructions. Briefly, after deparaffinization, incubated in Bouin's fixative at 60°C for 1 h. The slides were washed and stained in Weigert's Iron Hematoxylin working solution for 10 min. The slides were washed and stained in Biebrich Scarlet‐Acid Fuchsin solution for 5 min. The slides were washed and stained in phosphotungstic/phosphomolybdic acid for 10 min. Slides were stained with aniline blue for 5 min, washed, and transferred to 1% acetic acid for 5 min. All the slides were mounted and evaluated under a Leica microscope equipped with Leica DFC310 FX digital colour camera (Leica Microsystem). The images were quantified using QuPath as previously described.^48^

### 4.6 Human cell lines

Established human MLiM cell lines (JK‐0346‐ME, RW‐1211‐ME) from SJCI were attained from MLiM patients who received elective surgery. Established human MLNM cell lines (MH‐0331‐ME, SG‐0035‐ME) and MBM cell lines (M16, M20) from SJCI were attained from melanoma patients who received elective surgery. Established human PLiM (Capan‐1 and CFPAC1) cell lines were obtained from the American Type Culture Collection (ATCC). The above‐mentioned cell lines were cultured in RPMI‐1640 and supplemented with 10 mM HEPES, 10% heat‐inactivated fetal bovine serum (FBS), and 1% penicillin–streptomycin. An established human normal liver hepatocyte THLE‐2 cell line was obtained from ATCC. THLE‐2 was cultured in bronchial epithelial cell basal medium and supplemented with 10 mM HEPES, 10% FBS, 1% penicillin‐streptomycin, and a growth factor BulleKit (Lonza/Clonetics Corporation). All experiments were performed with mycoplasma‐free cell lines.

### 4.7 Cell viability assay

Normal liver hepatocyte cell lines (2 × 10^3^) were seeded in 96‐well culture plates. The number of viable cells was assessed using a Cell Titer‐Glo Luminescent Cell Viability assay (Promega) according to the manufacturer's instructions as previously described.^14^, ^47^

### 4.8 Cell line conditioned medium collection from MLiM, PLiM, and CLiM

MLiM cells (5 × 10^5^) were seeded in a 10 cm dish and incubated in 10 mL cultured medium. The culture medium was collected after 48 h incubation and centrifuged (4000×*g*, 15 min). A total of 9 mL of supernatant was collected. The supernatant was further centrifuged (12 000×*g*, 5 min). The supernatant was aliquoted and stored at −30°C until used for experiments.

### 4.9 RNA isolation and RT‐qPCR

Total RNA from cell lines was extracted by the Direct‐zol RNA miniprep kit (#R2050, Zymo Research) according to the manufacturer's instructions. Reverse transcription‐quantitative polymerase chain reaction (RT‐qPCR) was performed as previously described.^14^ Primer sets (Integrated DNA Technologies) used in RT‐qPCR are shown in Table S4.

### 4.10 Plasmids

Knockdown experiments were performed as previously described.^47^ Normal hepatocyte cell line THLE‐2 was transfected with 50 nM pool siRNA targeting CXCR4 or non‐targeting control (L‐005139‐00‐0005 and D‐001810‐10‐05, respectively, Horizon Discovery) using jetPRIME transfection reagent (Polypus‐transfection).

### 4.11 Nuclear extraction

Nuclear and cytoplasmic fractions were isolated from the THLE‐2 cell line with the nuclear extract kit (Active Motif, Carlsbad). Cells were cultured in 10 cm dishes and harvested with 3 mL cold PBS/Phosphatase inhibitor buffer. Cells were centrifuged and the whole‐cell pellet was gently suspended in 500 µL hypotonic buffer and incubated for 15 min on ice. Then, 25 µL of detergent was added to induce cell lysis. After cell lysis, the cytoplasmic fraction (supernatant) was separated from the nuclear fraction (pellet) by centrifugation (30 s at 14 000×*g*). Then, the nuclear fraction (pellet) was resuspended in 50 µL of the complete lysis buffer and incubated with 2.5 µL detergent for 30 min on ice. The nuclear lysates were centrifuged for 10 min at 14 000×*g*. The nuclear fraction (supernatant) was then collected. Both nuclear and cytoplasmic fractions were analyzed by western blot.

### 4.12 Western blot

Traditional western blot was performed as previously described.^14^ The antibodies utilized are summarized in Table S2. All traditional western blot bands were visualized on an iBright FL 1500 Imager (Thermo Fisher Scientific), and all images were analyzed with ImageJ software (http://imagej.nih.gov/ij/). All the uncropped western blot images were included in Figures S16–S22.

### 4.13 Indirect immunofluorescence

Normal hepatocyte cell lines (5 × 10^3^) were seeded in 8‐well Falcon chambered culture slides (Thermo Fisher Scientific). The supernatant medium of MLiM cell lines was administrated, and cells were incubated for 48 hours, as previously described.^47^ Ab dilutions are shown in Table S2. Each slide was imaged using the Mantra Multispectral Imaging System (v1.0, Akoya Biosciences). The CXCR4, COL1A1, and p50 fluorescence intensities were quantitatively evaluated using a 40× magnifying image in Qupath software (v.0.3.2, Queen's University). Fluorescence intensity was quantified automatically in the nucleus and cytosol areas of each cell. The mean intensities in each condition were compared for statistical significance.

### 4.14 3D‐spheroid assay and paraffin‐embedding

Normal hepatocyte cell lines (THLE‐2, 1 × 10^4^) were suspended in 100 µL of medium and cultured using a 3D‐spheroid microplate (Corning Inc.) as described previously.^14^ The medium was replaced on day 2 and then every two days. When indicated, 40 µL of the supernatant obtained from the JK‐0346‐ME cell line (MLiM) was administered every time the medium was changed. After 7 days of culturing spheroids were embedded in paraffin to form a block. Briefly, the spheroids were washed with PBS and fixed with 4% paraformaldehyde (PFA) for 15 min. Then, 4% PFA solution was removed, and 100 mM glycine was added. After 10 min incubation the spheroids were washed with PBS. For embedding in agarose gel, 5 to 6 spheroids were carefully transferred in 1.5 mL tubes and resuspended in 50 µL pre‐warmed 2% agarose in PBS and incubated at 4°C for 30 min for agarose gel to solidify. The gel containing spheroids was transferred to biopsy cassettes and stored in 70% ethanol. After dehydration in 80%, 90%, and 100% ethanol, xylene, and paraffin infiltration, the spheroids were paraffin‐embedded and sliced 5 µm using Thermo Scientific Microm HM 325‐2 manual microtome (Thermo Fisher Scientific). All images were acquired using Mantra Multispectral Imaging System (Akoya Biosciences) as previously described.^46^

### 4.15 Enzyme‐linked immunoassay

MLiM and PLiM cell lines (2 × 10^5^) were seeded in a six‐well plate and incubated for 24 h as described previously.^49^ TNF‐ α  levels in the supernatant were quantified using the TNF‐ α  Human ELISA kit (Thermo Fisher Scientific) according to the manufacturer's instructions.

### 4.16 Biostatistical analysis

All the biostatistical analyses were performed using NGDSP software, or R 4.2.1 version in a two‐tailed way. The distribution and variation within each group of data were assessed before selecting the correct statistical analysis. Fisher's exact test or Chi‐square test was performed for comparison with nominal variables. The student's *t*‐test or linear mixed model was used for comparison between the two groups. The correlation was determined by Spearman's correlation test. All data were presented as mean ± standard deviation (SD). *p*‐values < .05 were considered significant. *p < .05, **p < .01, and ***p < .001 was indicated as statistically significant. All figures were unified using Adobe Illustrator CC (Adobe) or CorelDraw graphics suite 8X (Corel).

## AUTHOR CONTRIBUTIONS

Shodai Mizuno designed the study, conducted experimental work, acquired and analyzed data, and wrote the first manuscript draft. Matias A. Bustos designed the study, conducted experimental work, acquired and analyzed data, and wrote the first manuscript draft. Yoshinori Hayashi, Kodai Abe, and Satoru Furuhashi conducted experimental work and acquired and analyzed data. Yalda Naeini and Xiaowei Xu contributed to FFPE selection and histopathological analysis. Anton J Bilchik contributed to patient recruitment and collected specimens and clinical data. Dave S. B. Hoon helped with funding acquisition, designed the study, and wrote the first manuscript draft. All authors reviewed, edited, and approved the final version of the manuscript.

## CONFLICT OF INTEREST STATEMENT

The authors declare no conflict of interest.

## ETHICS STATEMENT

The study was conducted following the Declaration of Helsinki. Human samples and clinical information for this study were obtained according to the protocol guidelines approved by the SJHC/SJCI Joint Institutional Review Board (IRB) and Western IRB: MORDRTPCR‐0995.

## Supporting information



Supporting information

Supporting information

Supporting information

Supporting information

Supporting information

Supporting information

Supporting information

## Data Availability

The NGDSP datasets for MLiM tissue samples supporting the conclusions of this article are available at GSE255328.
