# Supplementary material for: Induced collagen type‐I secretion by hepatocytes of the melanoma liver metastasis is associated with a reduction in tumour‐infiltrating lymphocytes
Source: Clin Transl Med. 2024 Nov 4;14(11):e70067. doi: 10.1002/ctm2.70067 (PMC11534464; doi:10.1002/ctm2.70067)
Supplement: Supplementary file 7 — Supporting information [file CTM2-14-e70067-s003.docx]

**Induced collagen type-I secretion by hepatocytes of the melanoma liver metastasis is associated with a reduction in tumor-infiltrating lymphocytes**

Shodai Mizuno^1^, Matias A. Bustos^1^, Yoshinori Hayashi^1^, Kodai Abe^1^, Satoru Furuhashi^1^, Yalda Naeini^2^, Xiaowei Xu^3^, Anton J. Bilchik^4^, and Dave S. B. Hoon^1, 5^

**Supporting Information**

**
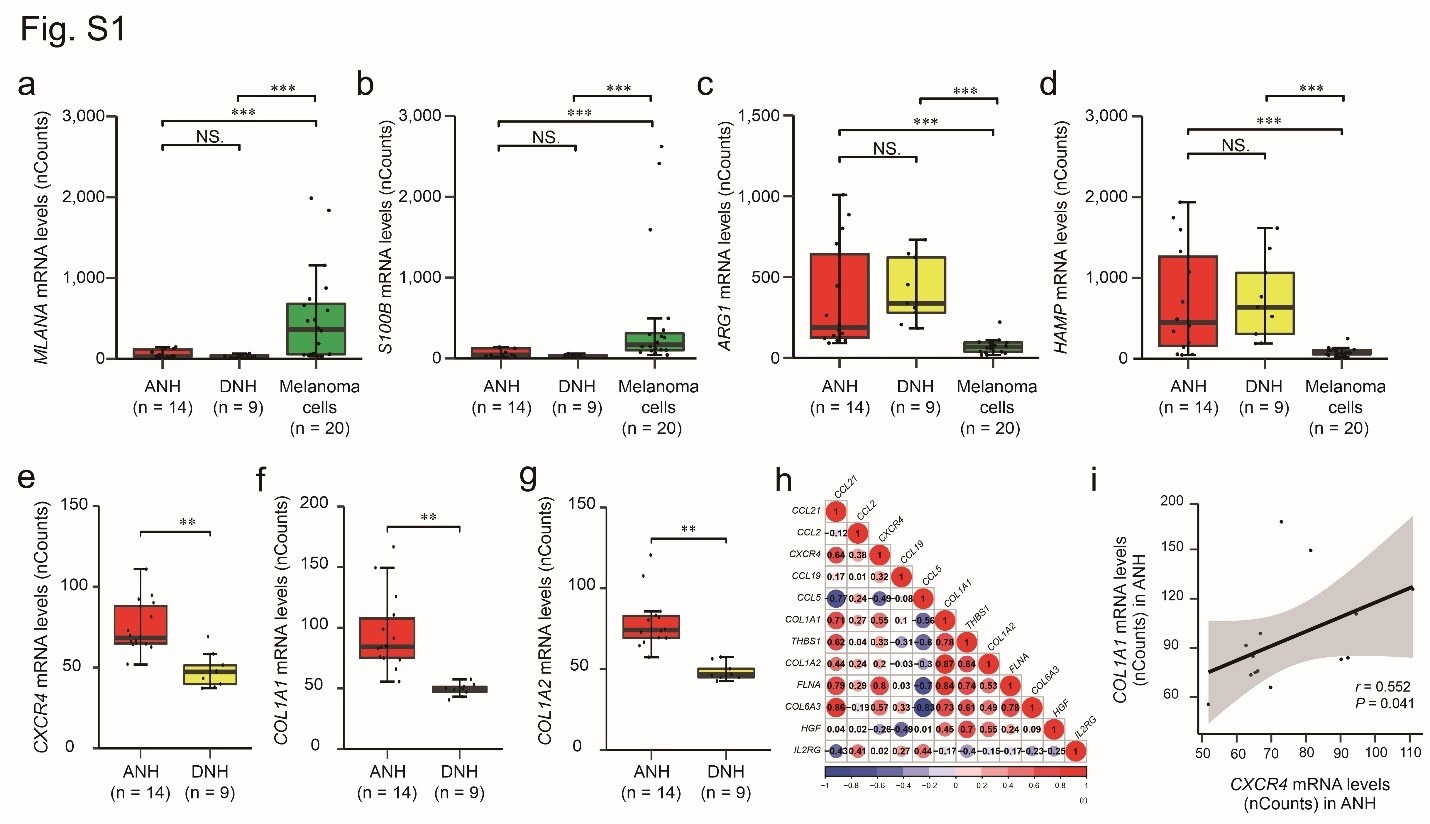
**

**Figure S1.** Spatial analysis in MLiM using NGDSP. **A-D** Box plot showing the mRNA levels of *MLANA* (**A**), *S100B* (**B**), *ARG1* (**C**), *HAMP* (**D**) in ANH, DNH, and melanoma cells. **E-G** Box plot showing the mRNA levels of *CXCR4* (**E**), *COL1A1* (**F**), and *COL1A2* (**G**) in ANH and DNH. **H** Correlation plot of common DEGs upregulated in ANH and chemokine signaling pathway, ANH and focal adhesion, and ANH and PI3K-Akt pathway. **I** Correlation between the mRNA levels of *CXCR4* and *COL1A1* in ANH. Data represent the mean ± SD. NS: not significant, ** *p* < 0.01, *** *p* < 0.001. The correlation was determined by Spearman’s correlation test **H**.


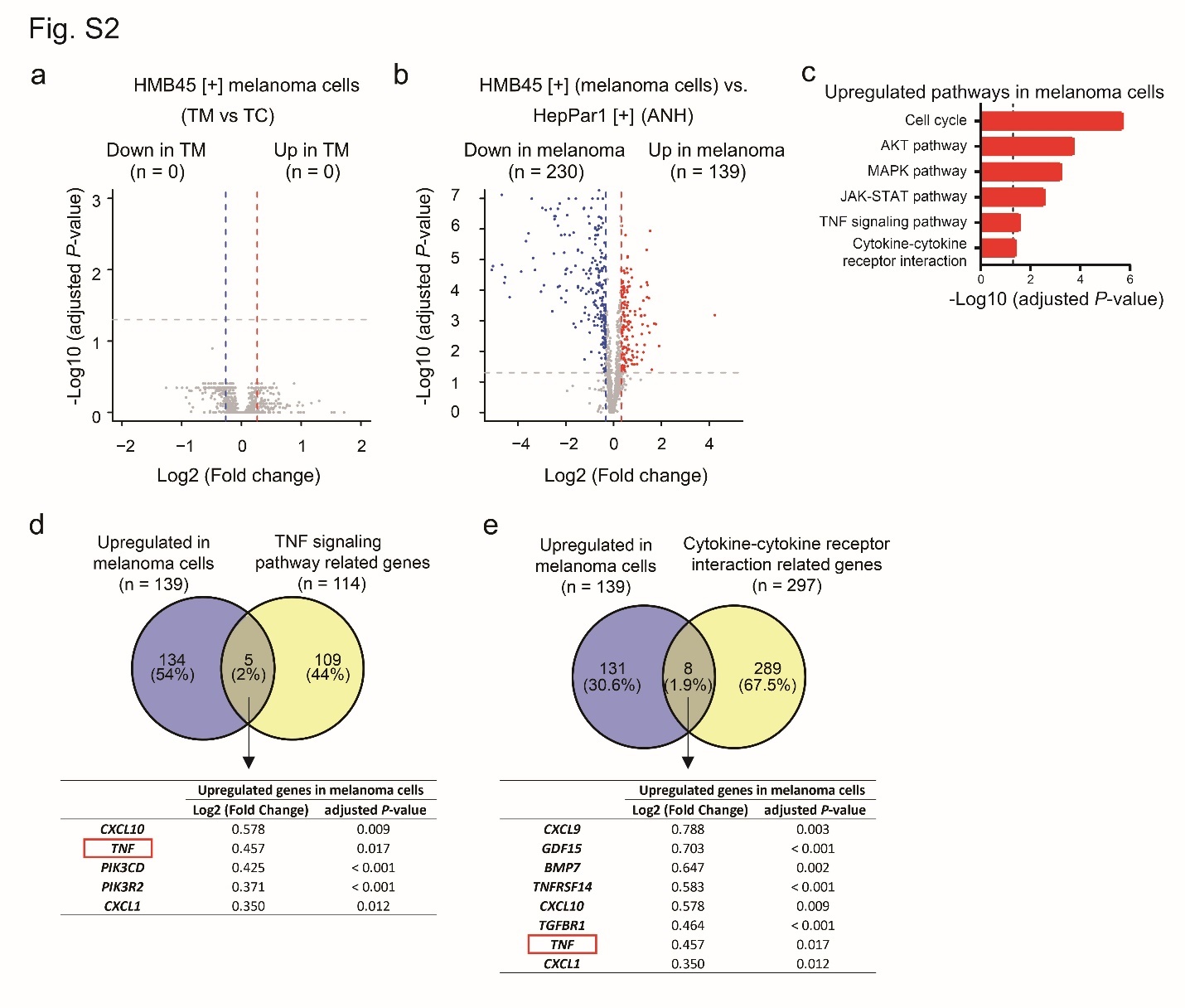


**Figure S2.** Spatial analysis in MLiM using NGDSP. **A** Volcano plot comparing HMB45[+] cells mRNA profiles between tumor margin (TM) and tumor center (TC). **B** Volcano plot showing the DEGs in HMB45[+] melanoma cells compared to HepPar1[+] adjacent normal hepatocytes (ANH) by NGDSP analysis. Of 369 DEGs, 139 were upregulated (red dots) and 230 were downregulated (blue dots) in melanoma cells. **C** Bar graph showing upregulated pathway in HMB45[+] in melanoma cells by KEGG pathway analysis. **D** Venn diagram showing the overlapping genes between the upregulated genes in melanoma cells and TNF signaling pathway related genes. The Table shows the genes common to the two groups. **E** Venn diagram showing the overlapping genes among the upregulated genes in melanoma cells and cytokine-cytokine receptor interaction related genes. The Table shows the genes common to the two groups.

**
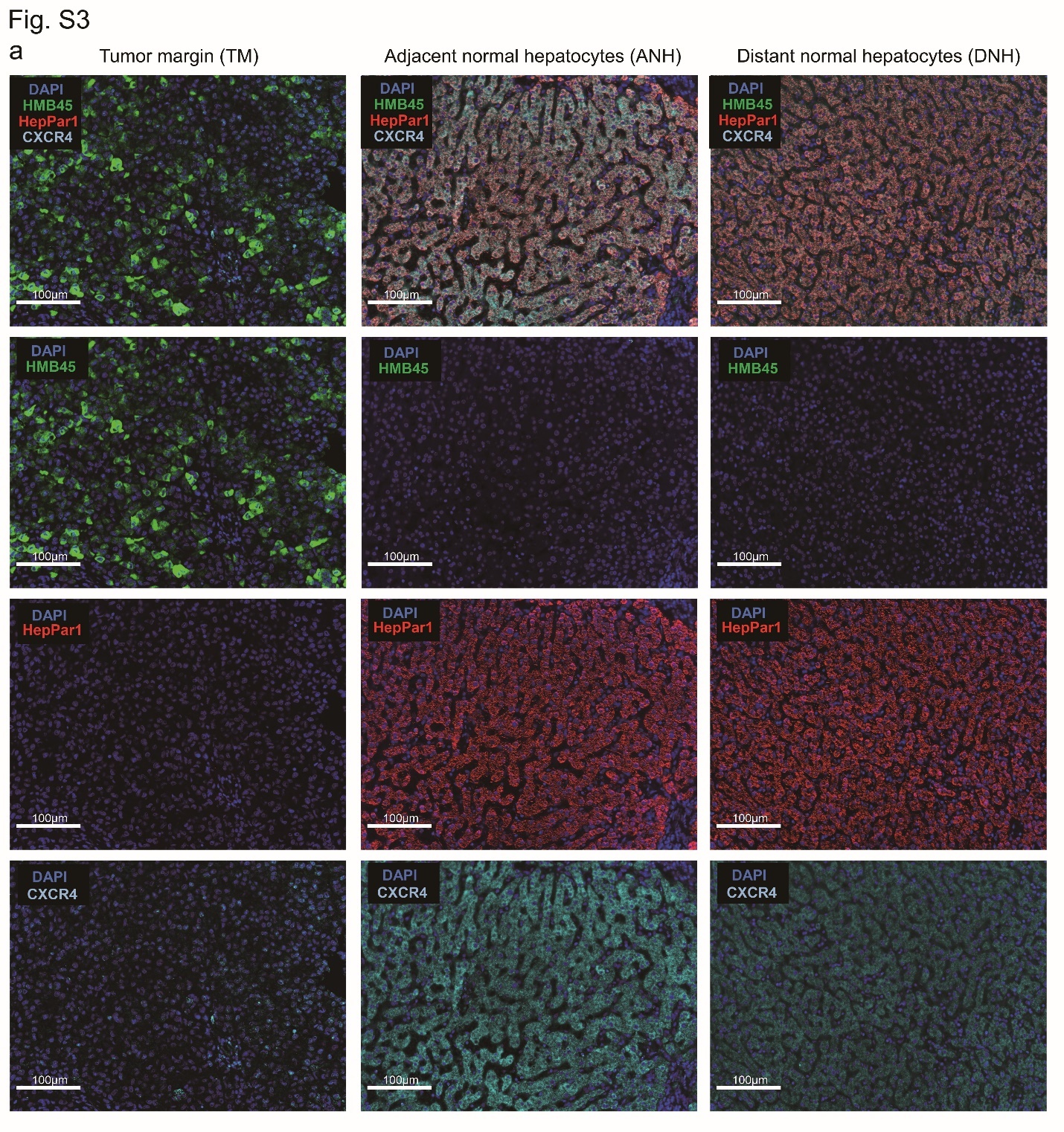
**

**Figure S3.** Representative multiplex immunofluorescence images of CXCR4 staining patterns of MLiM. **A** Representative image of the histological areas (Tumor margin (TM), adjacent normal hepatocytes (ANH), and distant normal hepatocytes (DNH)) in MLiM FFPE tissue biopsies that were stained using Opal mIF assay. Scale bar = 100 µm.


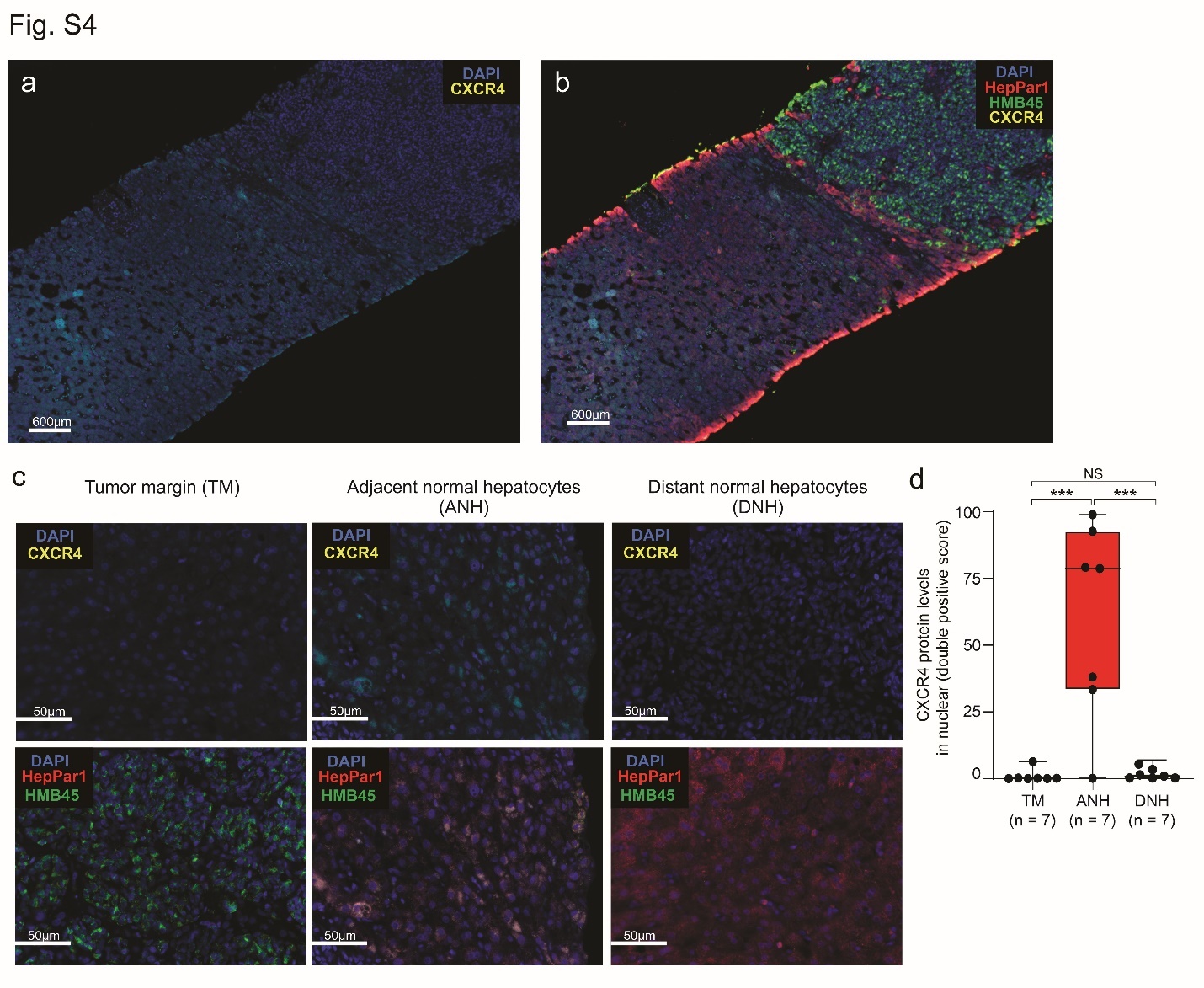


**Figure S4.** Representative multiplex immunofluorescence images of COL1A1 staining patterns of MLiM. **A** Representative image of the histological areas (Tumor margin (TM), adjacent normal hepatocytes (ANH), and distant normal hepatocytes (DNH)) in MLiM FFPE tissues that were stained using Opal mIF assay. Scale bar = 100 µm.


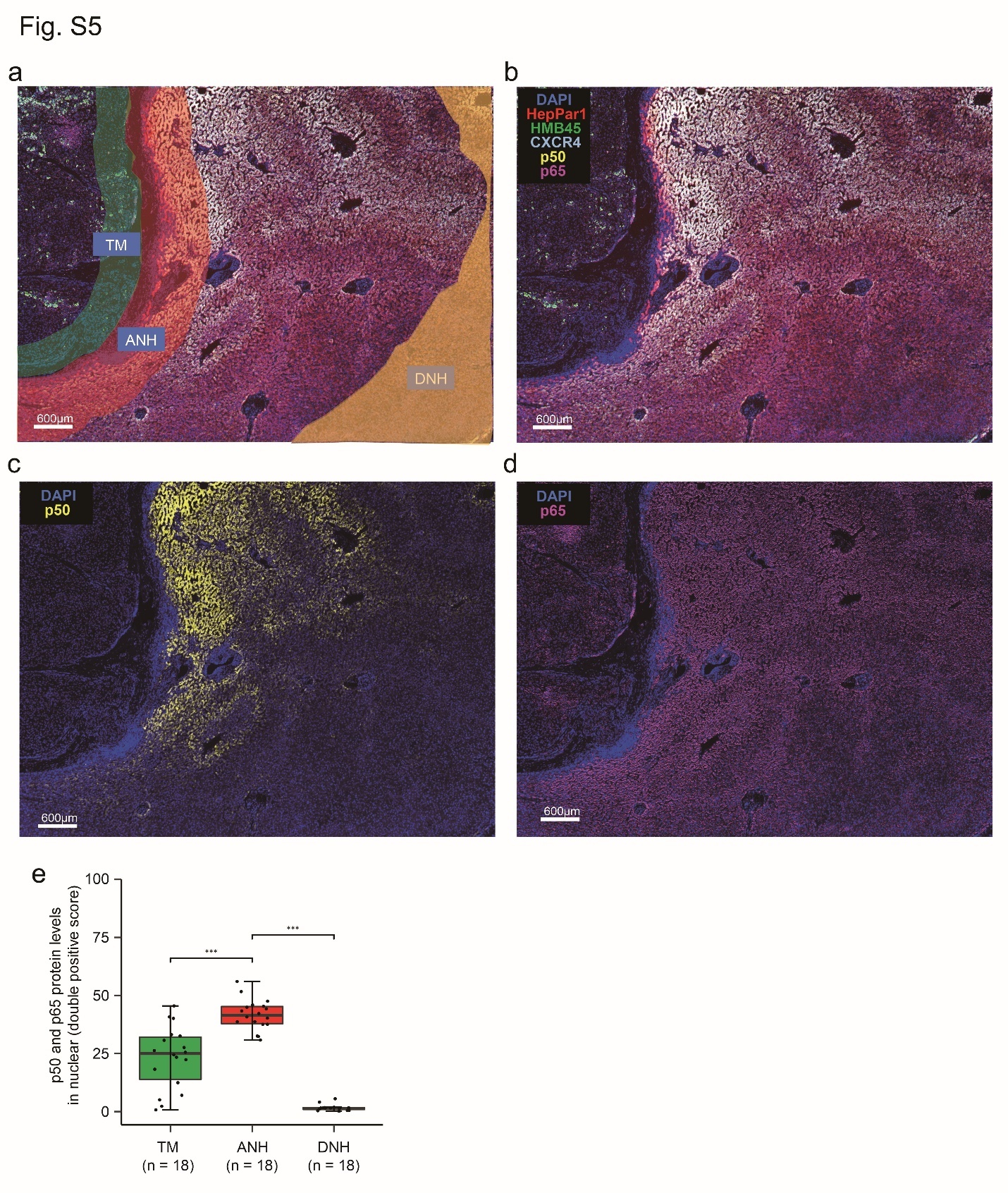


**Figure S5.** Representative multiplex immunofluorescence (mIF) images showing p50 and p65 staining patterns in MLiM. **A** Image showing the histological definition of tumor margin (TM), adjacent normal hepatocytes (ANH), and distant normal hepatocytes (DNH). **B-D** Representative mIF images of MLiM showing the merge image of DAPI/HepPar1/HMB45/CXCR4/p50/p65 (**B**), p50 (**C**), and p65 in FFPE tissue samples that were stained using Opal mIF assay. DAPI (blue); HMB45 (green); HepPar1 (red); CXCR4 (cyan); p50 (yellow); p65 (magenta). Scale bar = 600 µm. **E** Quantification of p50 and p65 protein levels in TM, ANH, and DNH. Data represents the mean ± SD. *** *p* < 0.001.


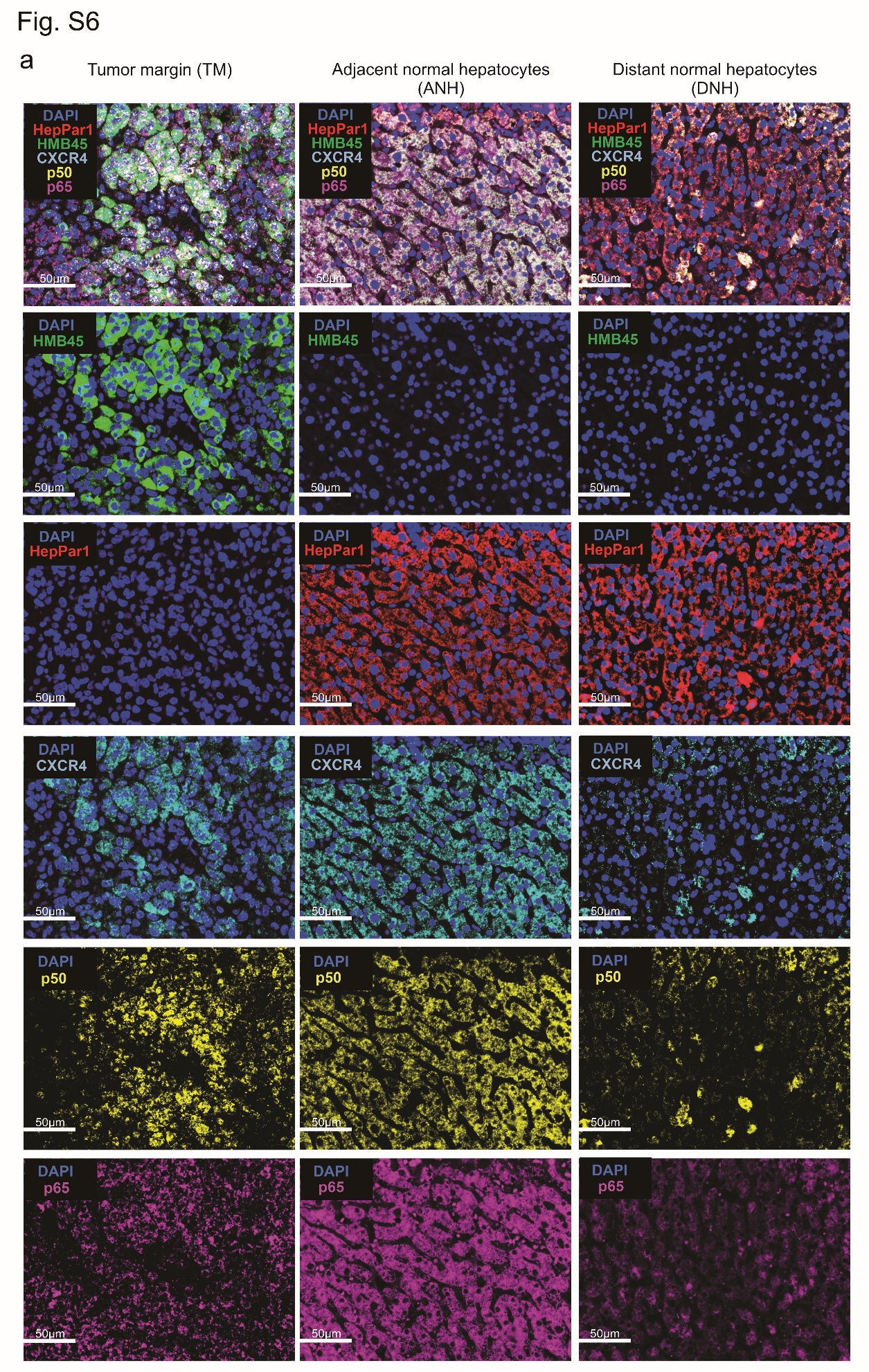


**Figure S6.** Representative multiplex immunofluorescence (mIF) images of CXCR4, p50 and p65 staining patterns in MLiM. **A** Representative image of the histological areas (Tumor margin (TM), adjacent normal hepatocytes (ANH), and distant normal hepatocytes (DNH)) in MLiM FFPE tissues that were stained using Opal mIF assay. Scale bar = 50 µm.


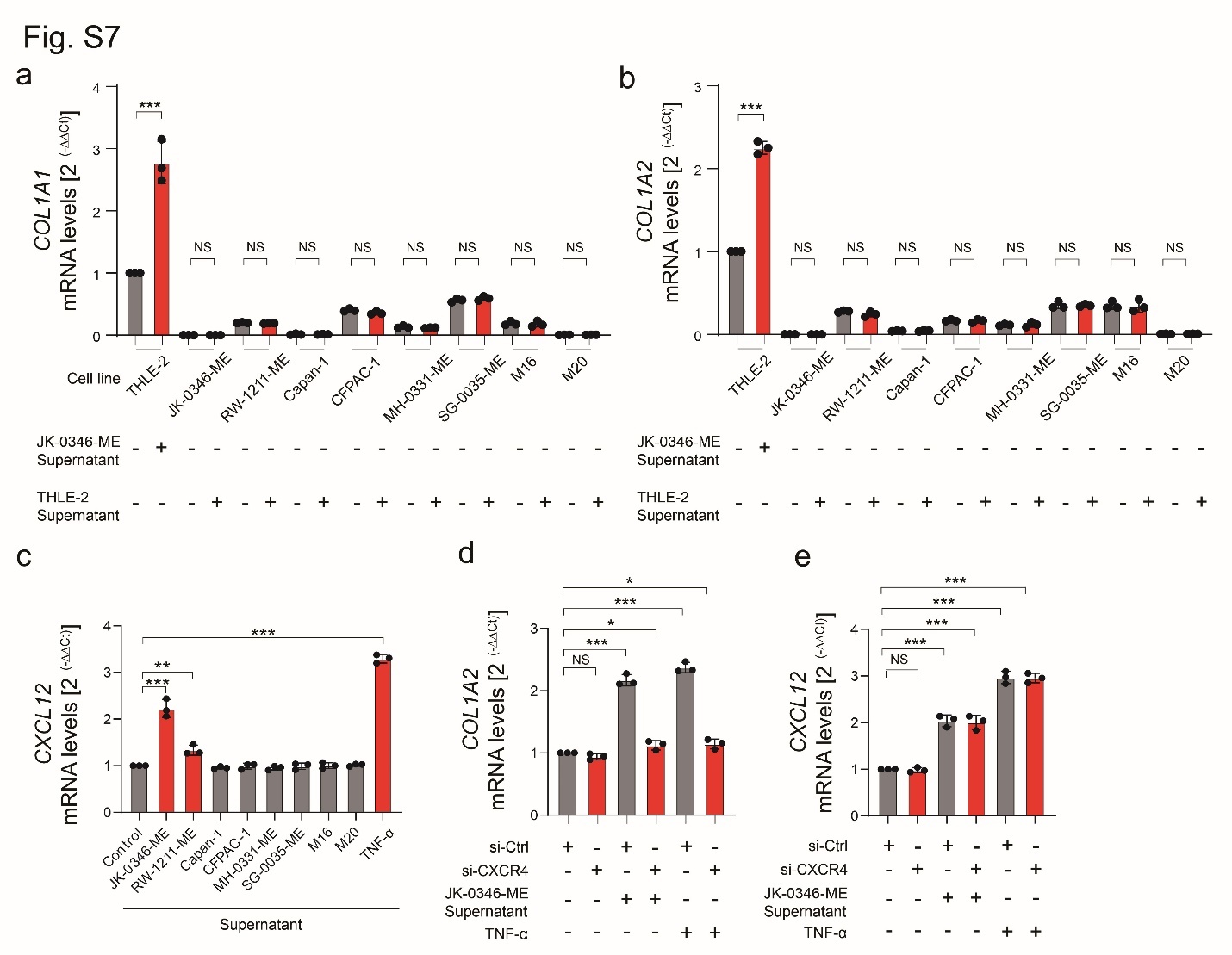


**Figure S7.** Analysis of THLE-2 cells treated with supernatant obtained from different cell lines. **A-B** Quantification of *COL1A1* (**A**) or *COL1A2* (**B**) mRNA levels in THLE-2 cells untreated or treated with MLiM supernatant, or in MLiM, PLiM, MLNM, or MBM cell lines untreated or treated with THLE-2 cell supernatant. **C** Quantification of *CXCL12* mRNA levels in THLE-2 cells treated with MLiM, PLiM, MLNM, or MBM supernatant or TNF-α treatment. **D-E** Quantification of *COL1A2* (**D**) *or CXCL12* (**E**) mRNA levels in THLE-2 after MLiM supernatant or TNF-α treatment in si-CXCR4 or si-Ctrl conditions. Data represent the mean ± SD. NS: not significant, * *p* < 0.05, *** *p* < 0.001.


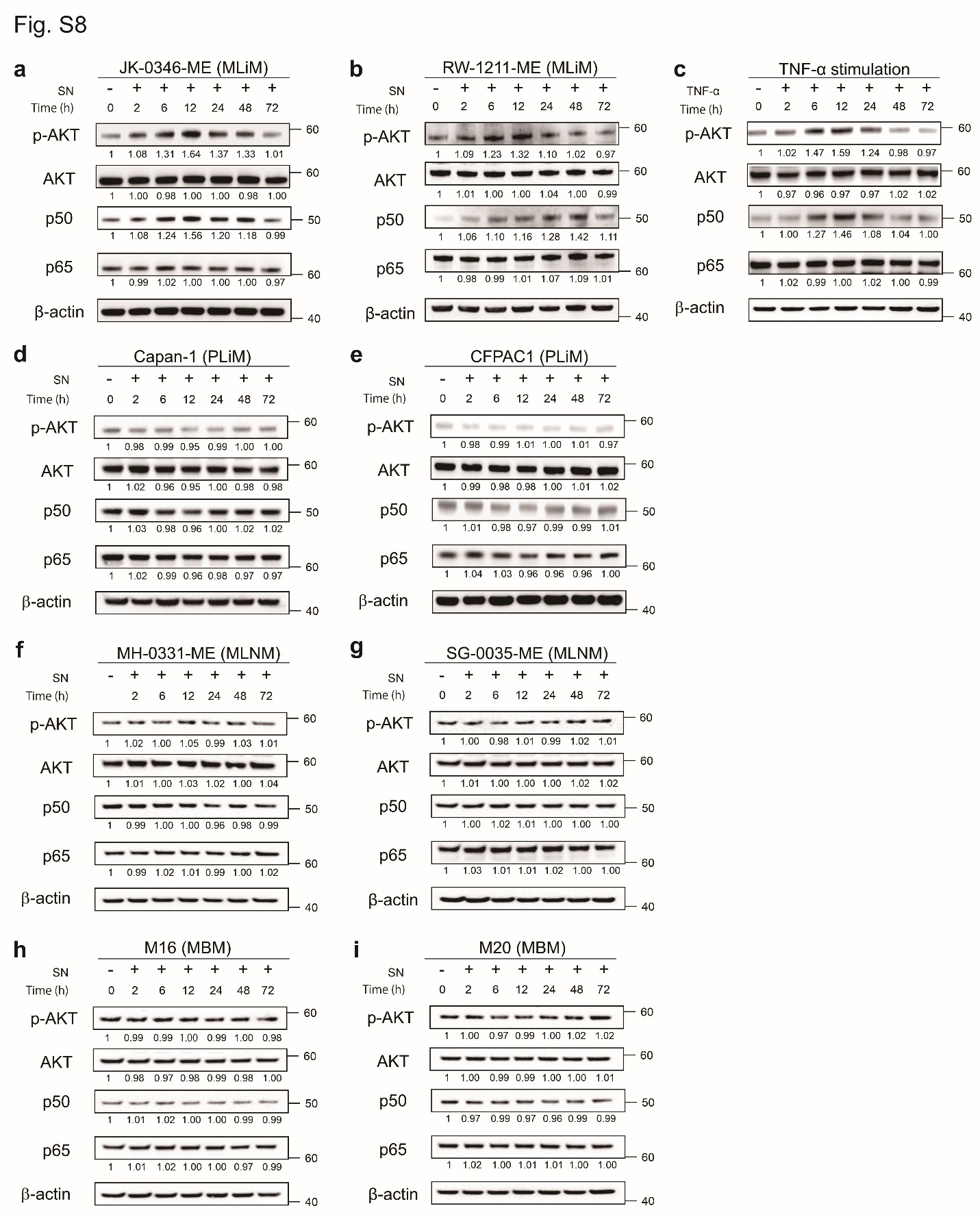


**Figure S8.** AKT and NFκB pathways are activated in THLE-2 cells incubated with MLiM, PLiM, MLNM, and MBM supernatants. **A-I** Western blot of p-AKT, AKT, p50, and p65 in THLE-2 cell lines after incubation with MLiM supernatant (**A, B**), or TNF-α (**C**), or PLiM supernatant (**D, E**), or MLNM supernatant (**F, G**), or MBM supernatant (**H, I**).


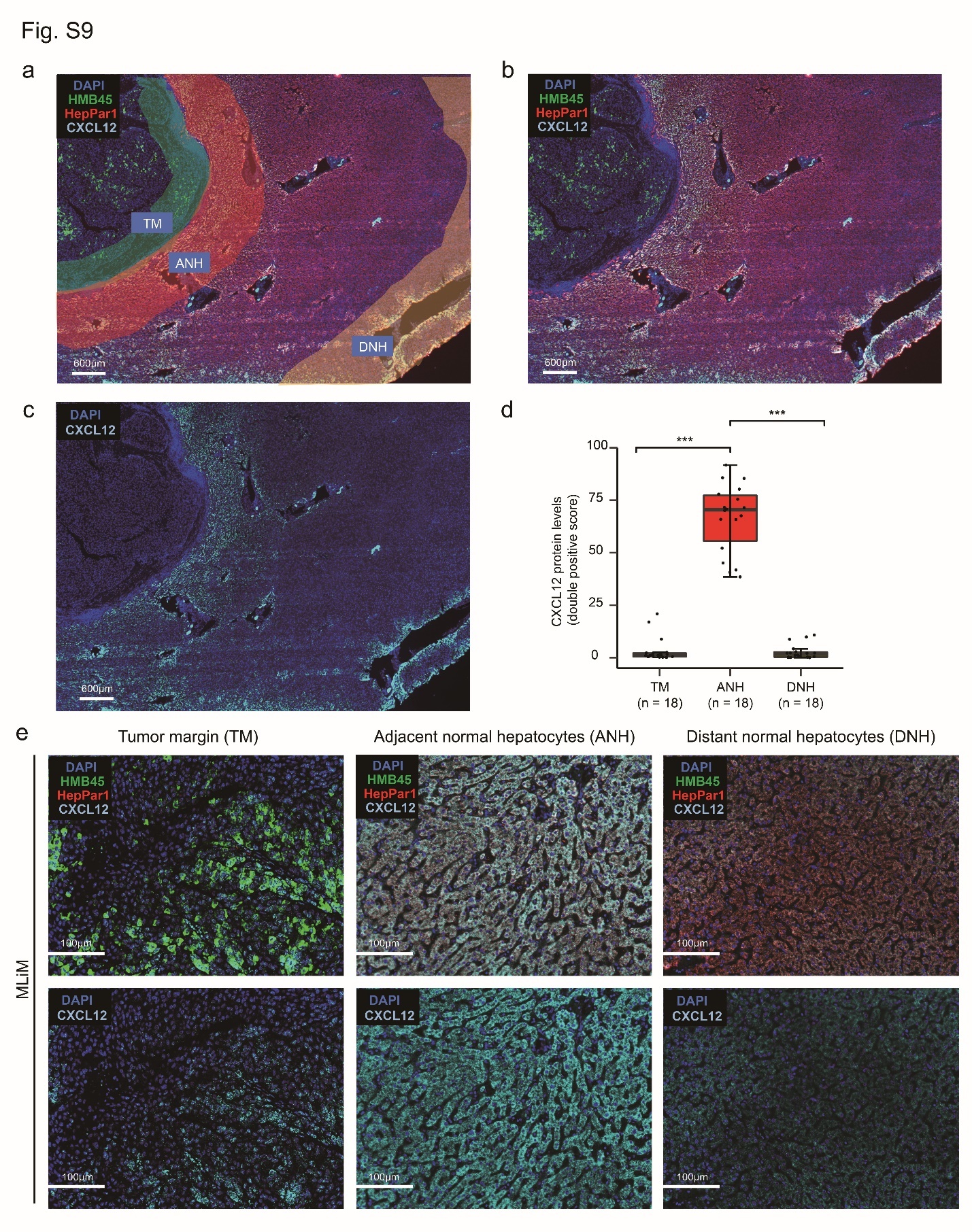


**Figure S9.** CXCL12 increased in ANH of MLiM. **A** Representative image of the histological areas (Tumor margin (TM), adjacent normal hepatocytes (ANH), and distant normal hepatocytes (DNH)) in MLiM FFPE tissues. **B-C** Representative mIF showing the merge image of (DAPI-HepPar1-HMB45-CXCL12, **B**) and CXCL12 (DAPI-CXCL12, **C**) that were stained using Opal mIF assay. DAPI (blue); HMB45 (green); HepPar1 (red); CXCL12 (cyan). Scale bar = 600 µm. **D** Quantification of CXCL12 protein levels in HepPar1[+] cells in ANH and DNH, and in HMB45[+] cells in TM. **E** Representative images of MLiM in TM, ANH, and DNH. Scale bar = 100 µm. Data represents the mean ± SD. *** *p* < 0.001.


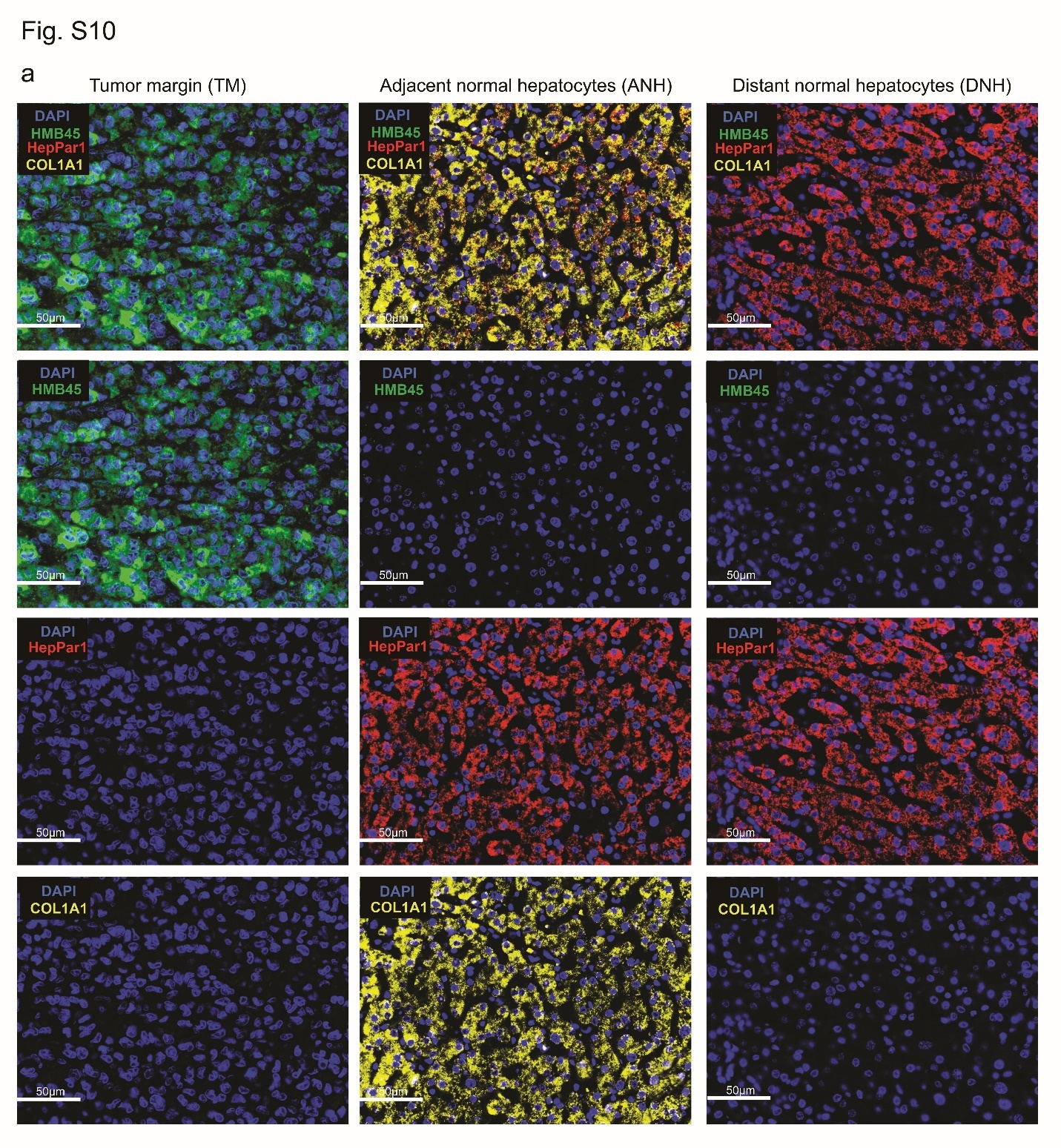


**Figure S10.** COL1A1 is elevated in ANH of MLiM. **A** Representative image of the histological areas (tumor margin (TM), adjacent normal hepatocytes (ANH), and distant normal hepatocytes (DNH)) in MLiM FFPE tissues that were stained using Opal mIF assay. Scale bar = 50 µm.

**
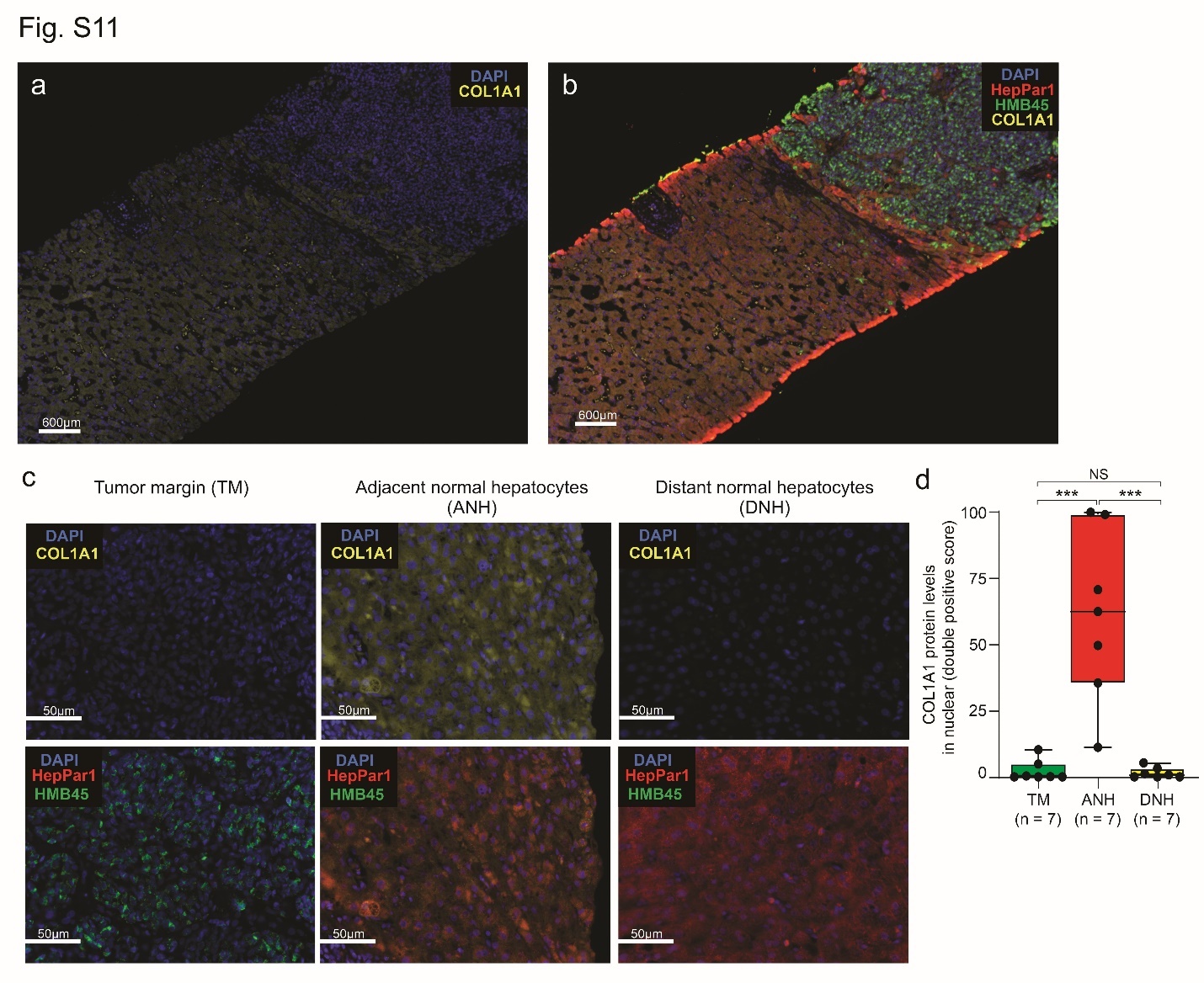
**

**Figure S11.** Representative multiplex immunofluorescence images of COL1A1 staining patterns of MLiM. **A** Representative image of the histological areas (Tumor margin (TM), adjacent normal hepatocytes (ANH), and distant normal hepatocytes (DNH)) in MLiM FFPE tissue biopsies that were stained using Opal mIF assay. Scale bar = 100 µm.


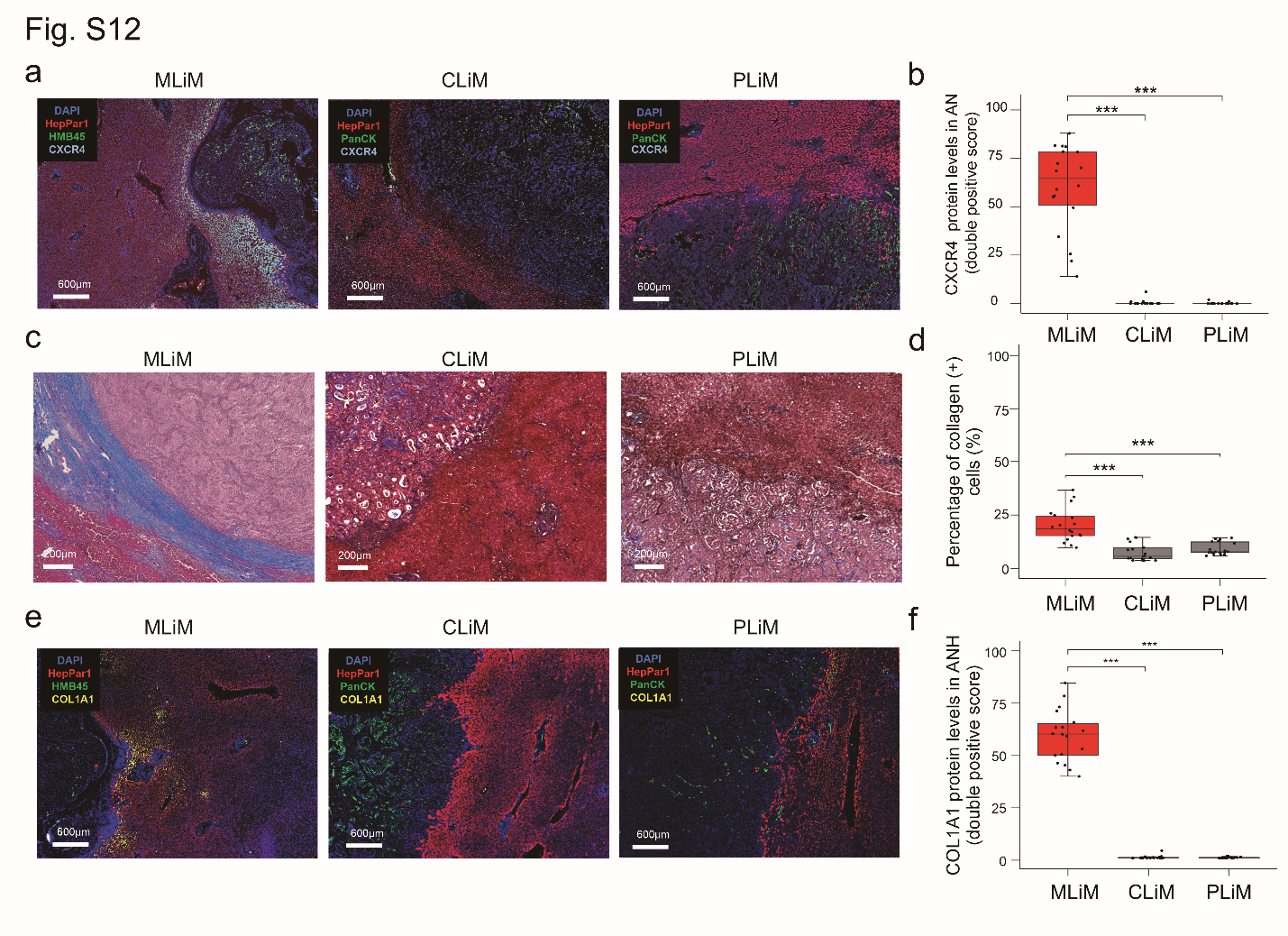
**Figure S12.** Comparison of CXCR4 and COL1A1 protein levels in ANH from MLiM, CLiM, and PLiM tissue s. **A** Representative mIF images of MLiM, CLiM, and PLiM FFPE tissue samples that were stained using Opal mIF assay. DAPI (blue); HMB45/PanCK (green); HepPar1 (red); CXCR4 (cyan). Scale bar = 600 µm. **B** Quantification of CXCR4 protein levels in HepPar1[+] cells in ANH. **C** Representative image of collagen staining of MLiM, CLiM, and PLiM. Scale bar = 200 µm. **D** Percentage of collagen positive cells of MLiM, CLiM, and PLiM. **E** Representative mIF image of MLiM, CLiM, and PLiM that were stained using Opal mIF assay. DAPI (blue); HMB45/PanCK (green); HepPar1 (red); COL1A1 (yellow). Scale bar = 600 µm. **F** Quantification of COL1A1 protein levels in HepPar1[+] cells in ANH. Data represents the mean ± SD. NS: not significant, *** *p* < 0.001.


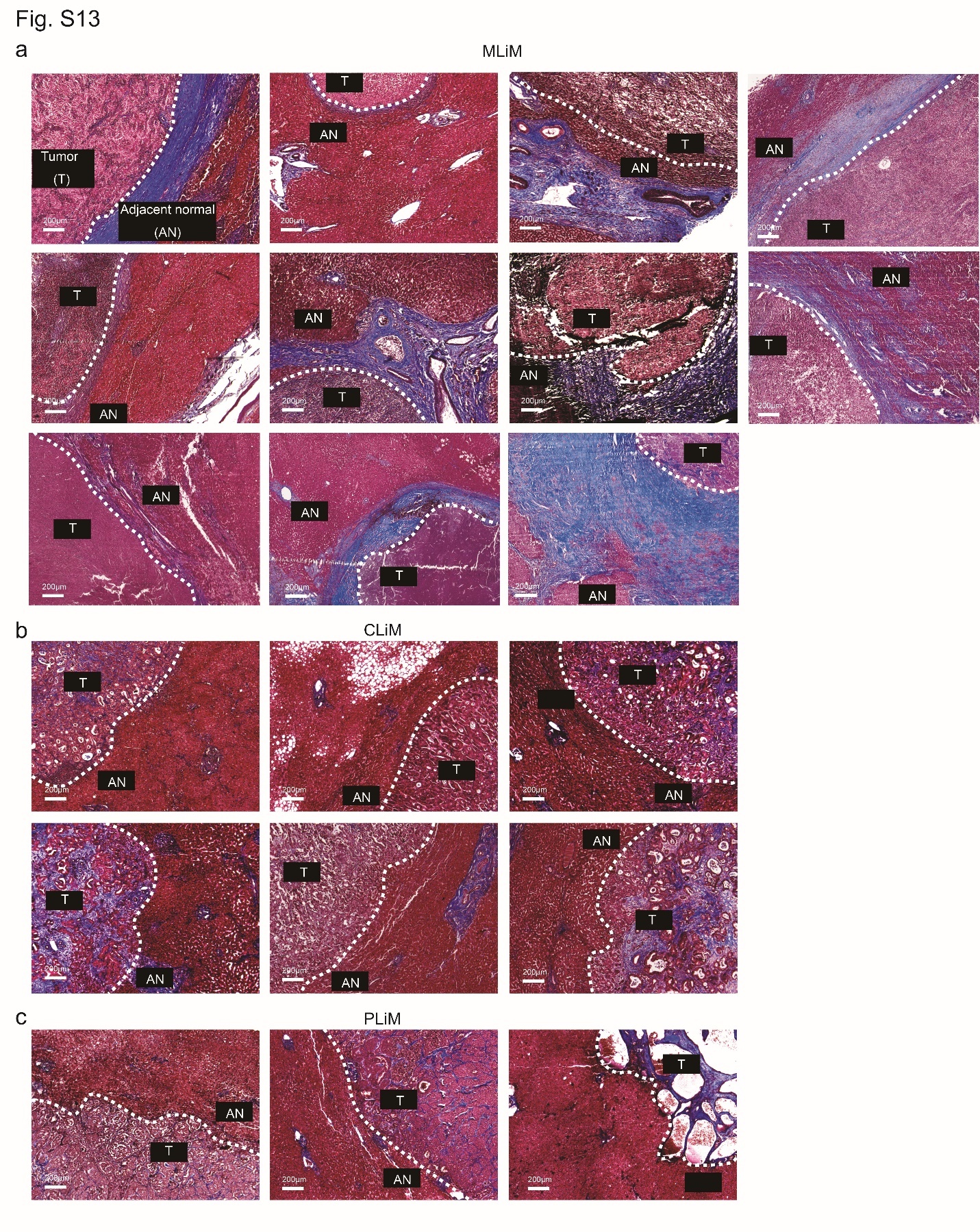


**Figure S13.** Collagen staining of all the liver metastasis analyzed. **A-C** Representative images of each MLiM (**A**), CLiM (**B**), or PLiM (**C**) FFPE tissue samples included in the study. Dotted line separates tumor (T) from adjacent normal (AN) areas. Scale bars = 200 µm.


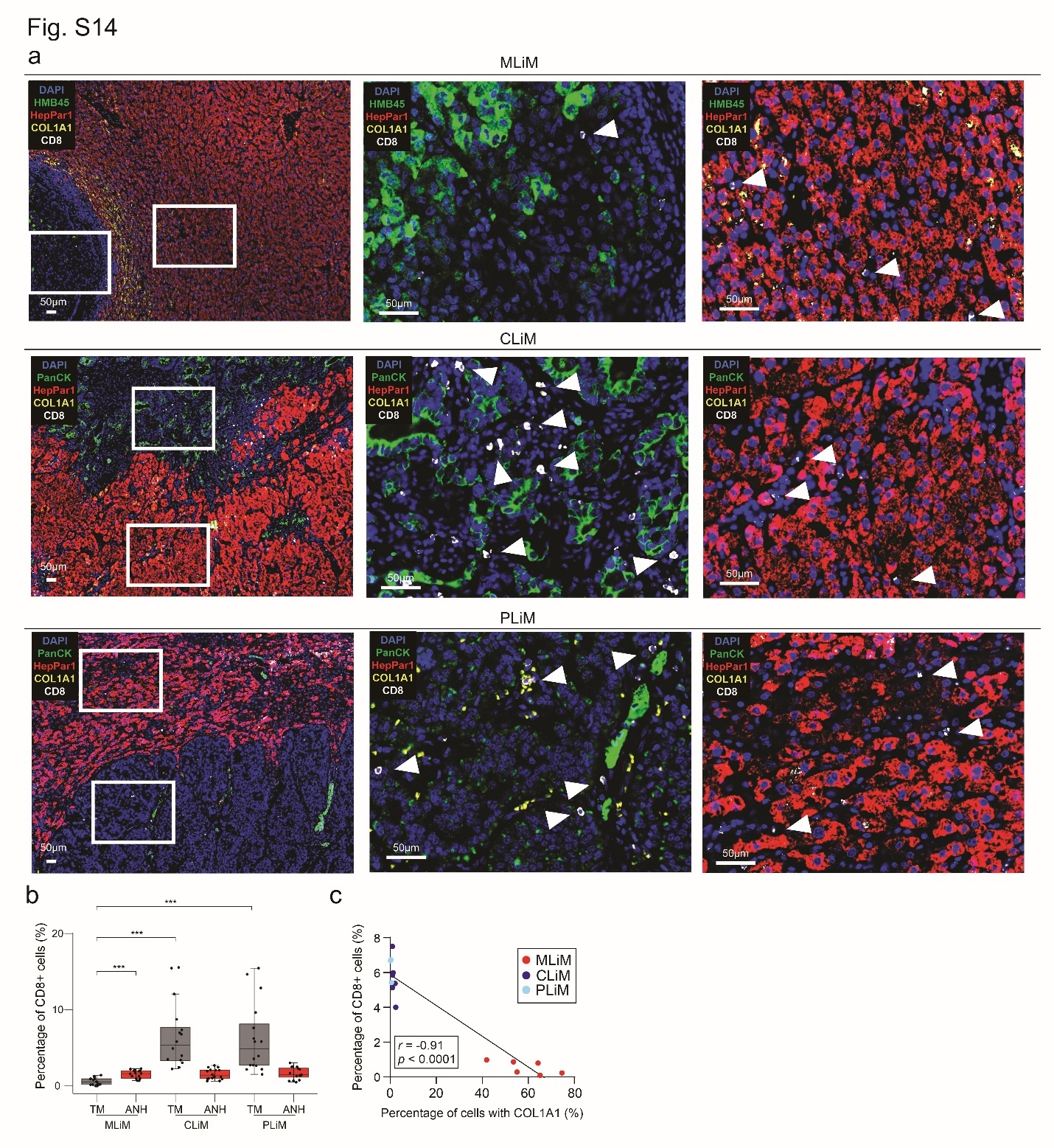


**Figure S14.** Percentage of CD8^+^ T cells in MLiM, CLiM, and PLiM. **A** Representative mIF images of MLiM, CLiM, and PLiM FFPE tissue samples that were stained using Opal staining assay. DAPI (blue); HMB45/PanCK (green); HepPar1 (red); COL1A1 (yellow); CD8^+^ (white). Scale bar = 50 µm. **B** Percentage of CD8^+^ cells in tumor margin (TM) vs. adjacent normal hepatocytes (ANH) of MLiM, CLiM, and PLiM. Data represents the mean ± SD. *** *p* < 0.001.


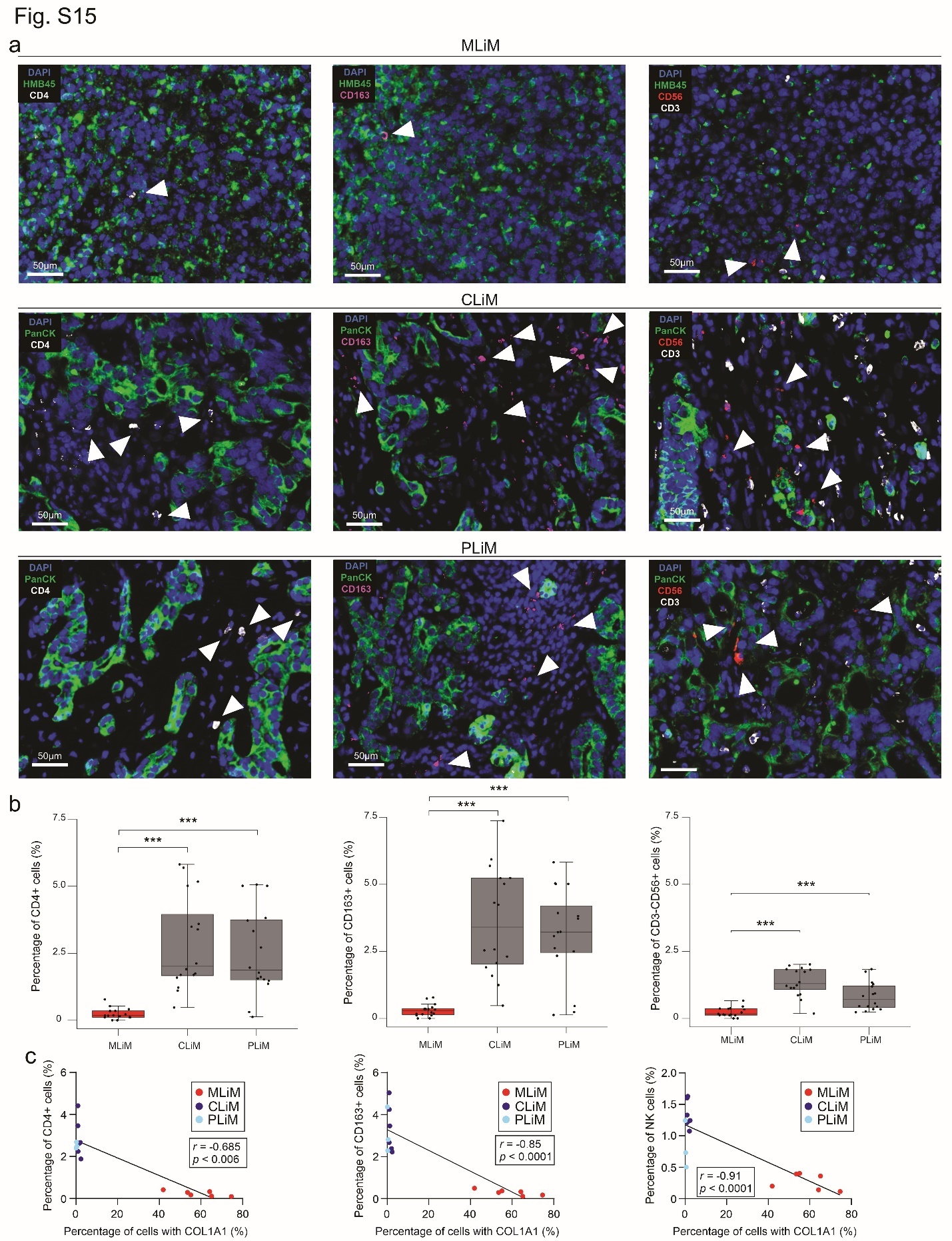


**Figure S15.** Percentage of CD4^+^ T cells, CD163^+^ macrophages, and CD3^-^/CD56^+^ NK cells in MLiM, CLiM, and PLiM. **A** Representative mIF image of MLiM, CLiM, and PLiM FFPE tissues samples that were stained using Opal mIF assay. DAPI (blue); HMB45/PanCK (green); CD4 (white); CD163 (magenta); CD3 (white); CD56 (red). Scale bar = 50 µm. **B** Percentage of CD4^+^ cells, CD163^+^ cells, and CD3^-^/CD56^+^ cells in tumor margin (TM) vs. adjacent normal hepatocytes (ANH) of MLiM, CLiM, and PLiM. Data represent the mean ± SD. *** *p* < 0.001.


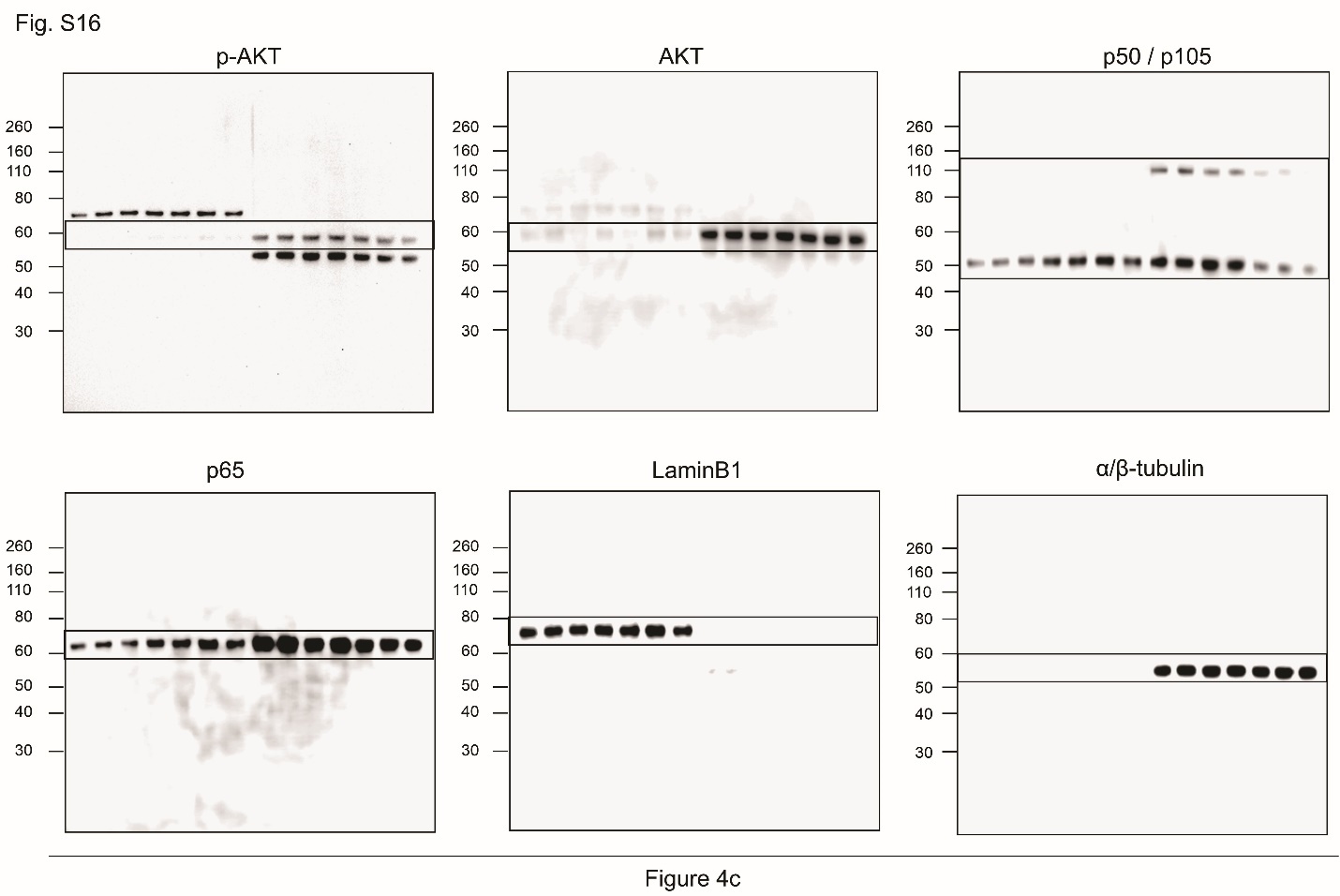


**Figure S16.** Uncropped western blot images. Uncropped western blot images for Figure 4C.


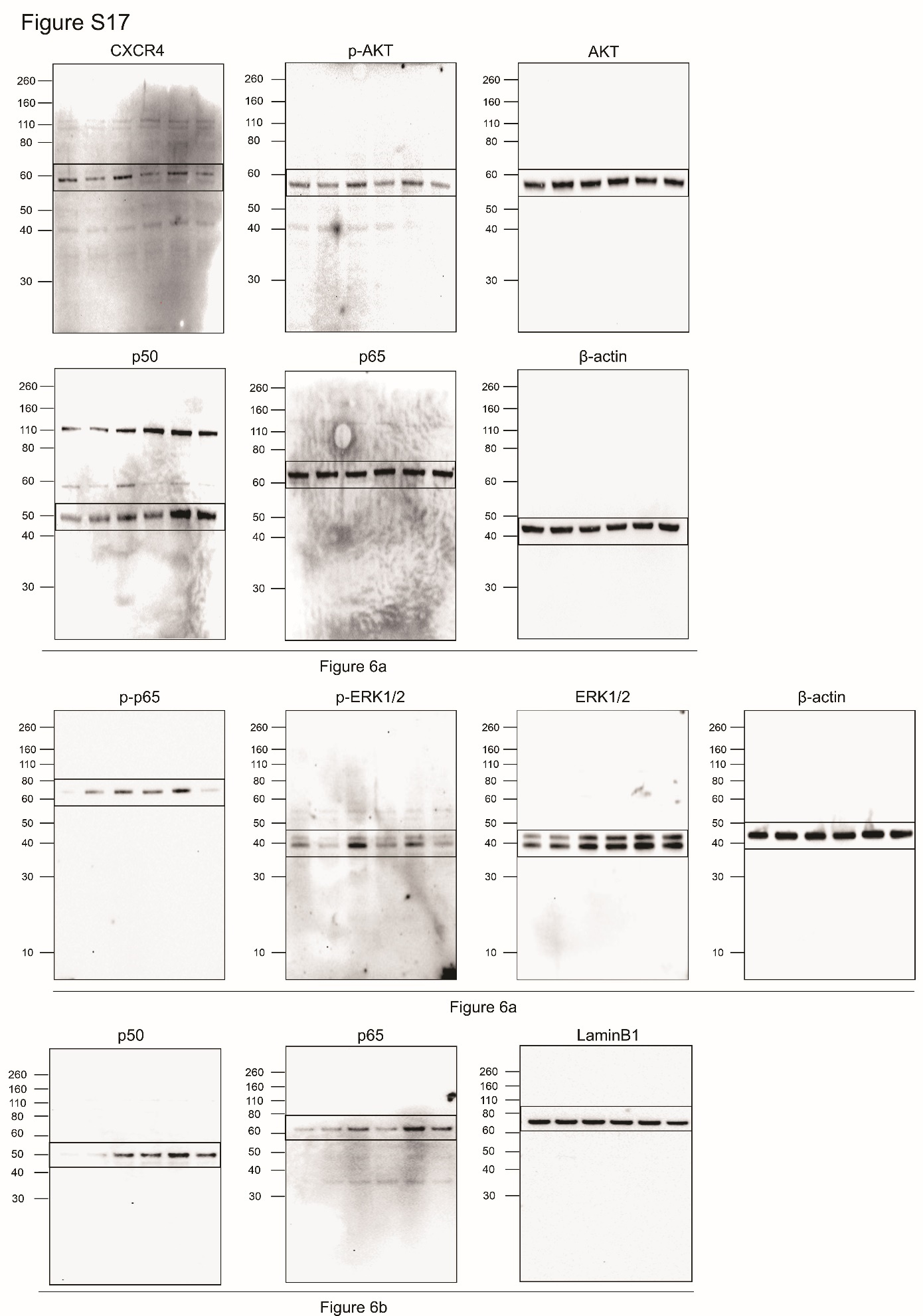


**Figure S17. Uncropped western blot images.** Uncropped western blot images for Figure 6A, B.


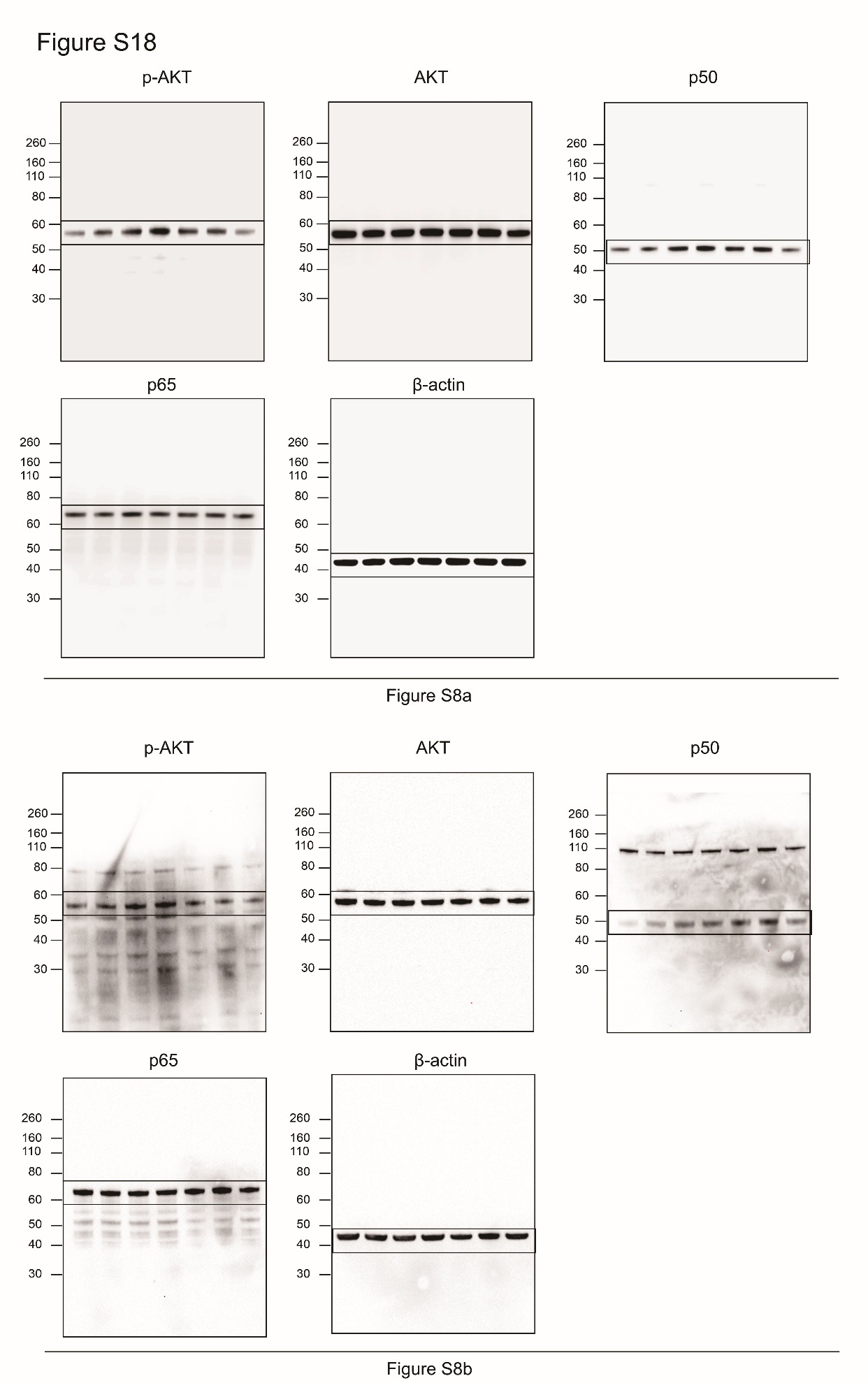


**Figure S18.** Uncropped western blot images. Uncropped western blot images for Figure S8A, B.


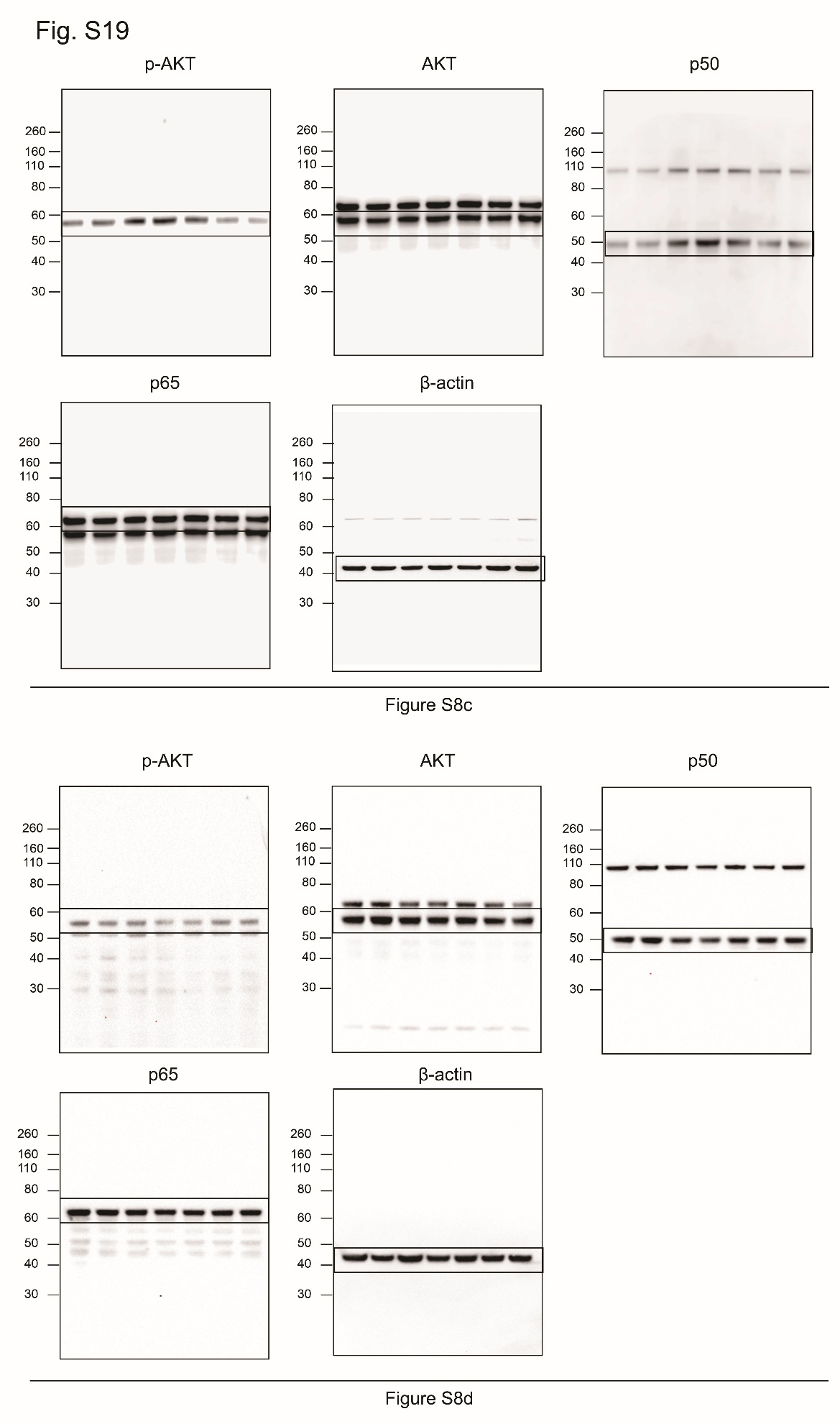


**Figure S19.** Uncropped western blot images. Uncropped western blot images for Figure S8C, D.


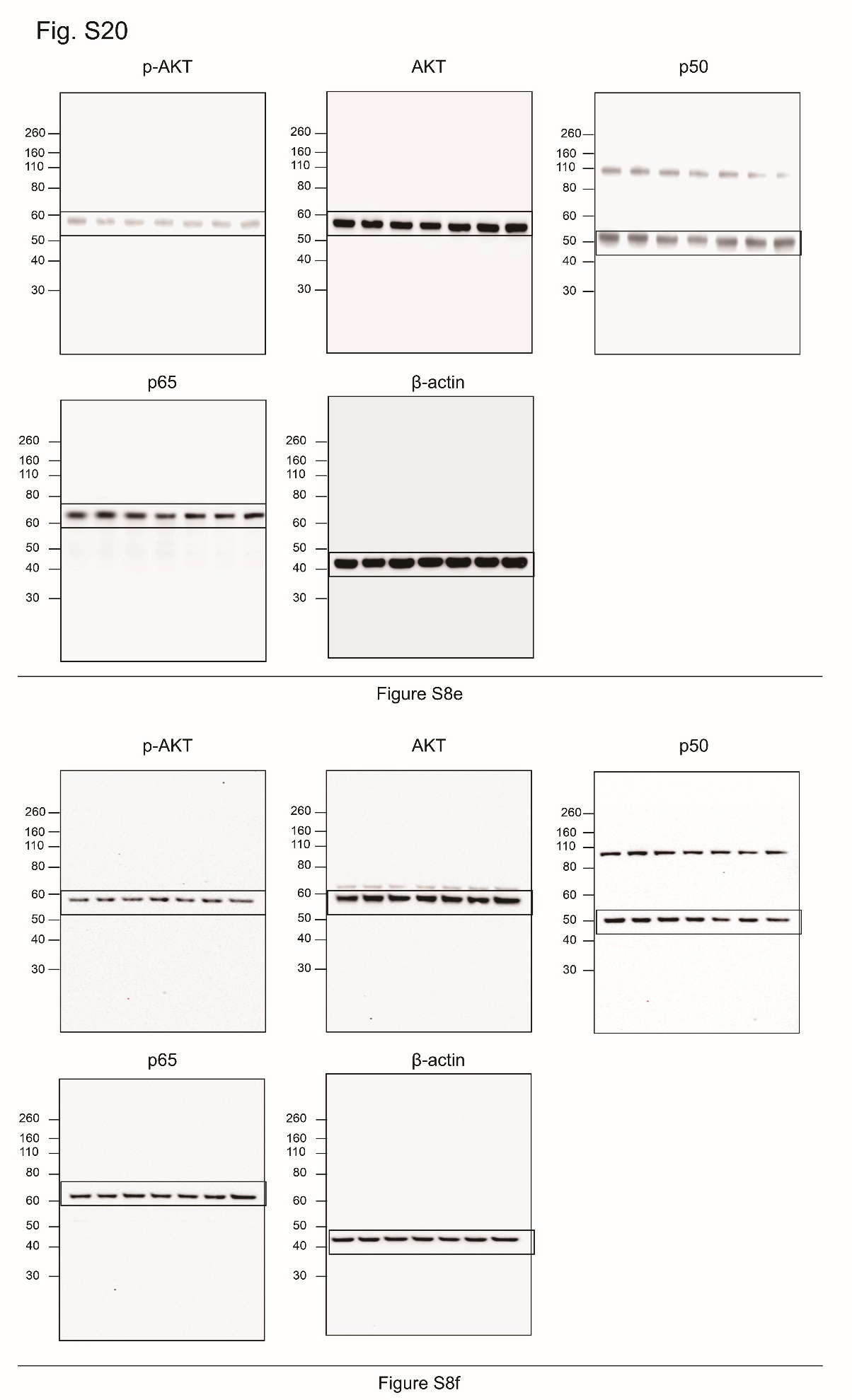


**Figure S20.** Uncropped western blot images. Uncropped western blot images for Figure S8E, F.


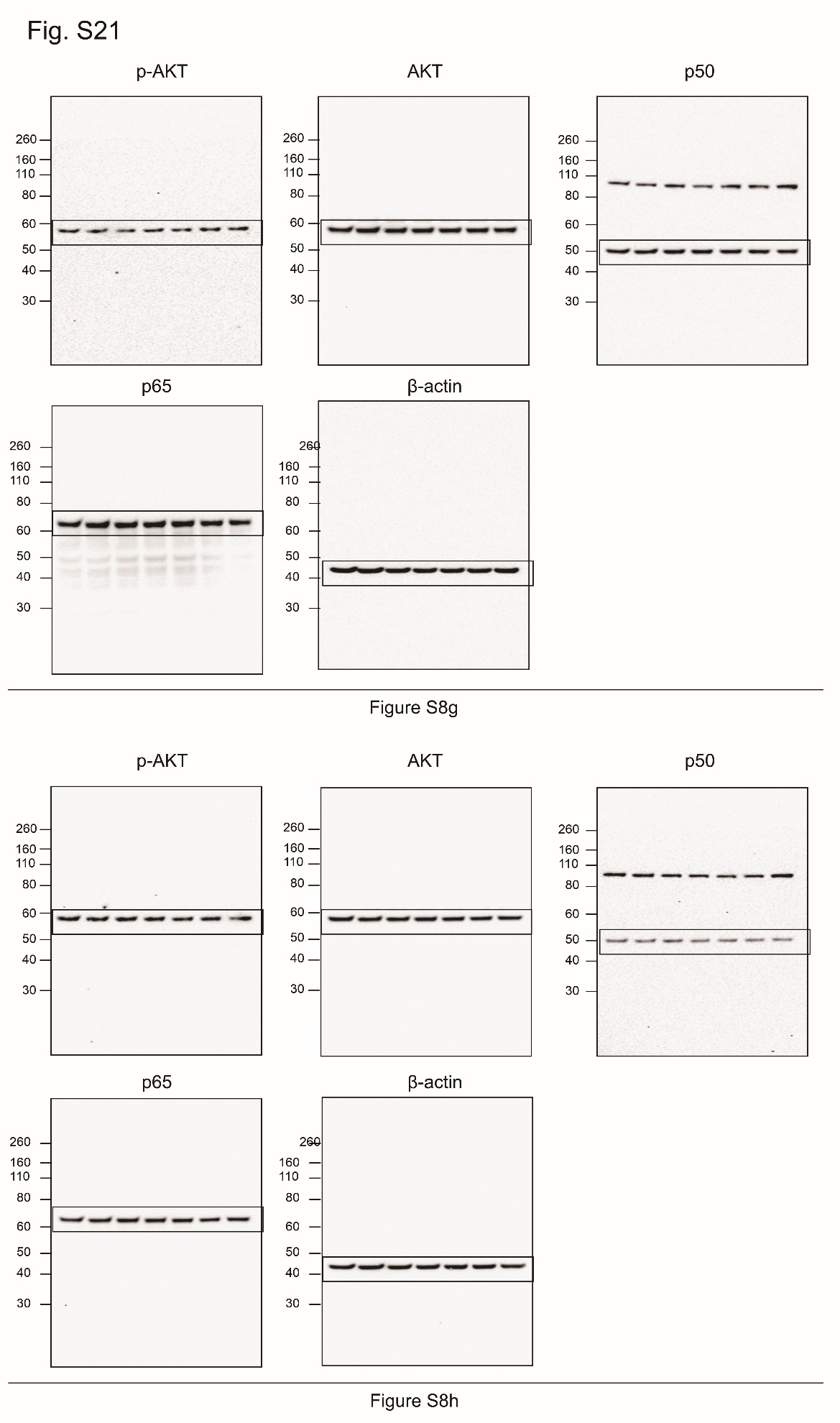


**Figure S21.** Uncropped western blot images. Uncropped western blot images for Figure S8G, H.


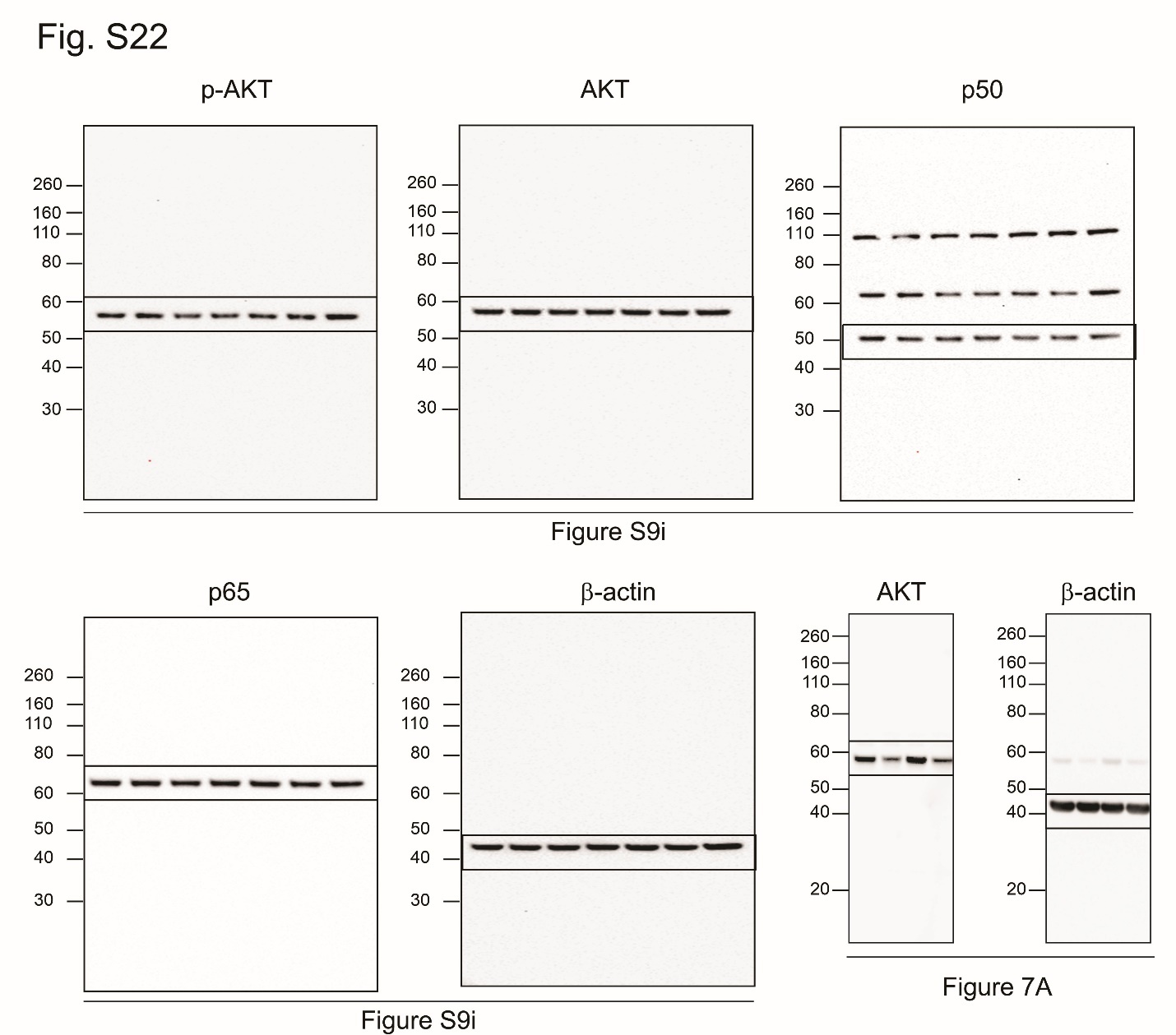


**Figure S22.** Uncropped western blot images. Uncropped western blot images for Figure 7A and Figure S8I.
